# Bioinspired Polymeric Scaffolds for Improvement of Angiogenesis and Tissue Engineering: A Review

**DOI:** 10.3390/polym18101224

**Published:** 2026-05-17

**Authors:** Vyas Jigar, Raytthatha Nensi, Vyas Puja, Bhupendra Prajapati, Pattaraporn Panraksa, Sudarshan Singh, Chuda Chittasupho

**Affiliations:** 1 Krishna School of Pharmacy and Research, Drs. Kiran & Pallavi Patel Global University, Varnama, Vadodara 391240, Gujarat, India; drjigarvyas@gmail.com (V.J.); nensiraytthatha@gmail.com (R.N.); 2 Neotech Institute of Pharmacy, Neotech Technical Campus, Virodh, Vadodara 390022, Gujarat, India; pujavyas20@gmail.com; 3 Department of Pharmaceutics, Parul Institute of Pharmacy, Faculty of Pharmacy, Parul University, Waghodia, Vadodara 391760, Gujarat, India; bhupen27@gmail.com; 4 Faculty of Pharmacy, Chiang Mai University, Chiang Mai 50200, Thailand; pattaraporn.pan@cmu.ac.th; 5 Office of Research Administration, Chiang Mai University, Chiang Mai 50200, Thailand

**Keywords:** angiogenesis, tissue regeneration, vascularization, endothelial signaling, bioinspired scaffolds, tissue engineering, regenerative medicine

## Abstract

Poor vascularization is one of the basic obstacles to the regeneration of functioning tissues because an oxygen diffusion process and elimination of wastes are essential in preserving the grafts. Recently, biomaterials have allowed the invention of bioinspired polymer scaffolds and replicated the natural extracellular matrix (ECM) due to the mechanical tunability of the synthetic polymers with the biological signals of natural macromolecules. The review uses a mechanistic analysis of the strategies to improve angiogenesis by using surface topography modification, bioactive peptide incorporation and pre-vascularization. Another way to achieve complex, perfusable topologies is by using more sophisticated methods of fabrication, such as electrospinning, 3D/4D bioprinting, or microfluidics. Based on in vitro and in vivo results, we determine angiogenic effectiveness by using cellular assays and animal transfers, pointing towards the translational advances in patents and clinical uses of bone, cardiac, nervous, and skin tissues. In spite of the substantial improvements, large-scale production and high demands of the regulations still exist. The future directions include the incorporation of bioinspired designs and intelligent materials, nanotechnology, and AI-based optimization into developing patient-specific and adaptive scaffolds. The following innovations herald the advent of highly effective constructs that can be used to regenerate tissue and overcome the limitations of present tissue engineering therapies through the introduction of highly effective, vascularized constructs.

## 1. Introduction

Tissue engineering is a multidisciplinary field devoted to the restoration and increase in the functionality of damaged biological tissues and organs. It integrates biomaterials, cells, and bioactive factors to develop living substitutes that resemble the natural tissue environment, stimulating biological processes such as cell attachment and tissue regeneration. Engineered scaffolds serve as three-dimensional structures that mimic the architecture and biochemical signals of the natural extracellular matrix (ECM) of the body, to assist cell adhesion and nutrient supply [1].

Tissue engineering (TE) and regenerative medicine [2] have been established as revolutionary disciplines to restore, maintain, or enhance tissue function by integrating scaffolds, cells, and biologically active molecules [3]. While great progress has been achieved in the fabrication of constructs for structural support, the long-term survival and integration of these engineered tissues is critically dependent on the establishment of a functional vascular network [4,5].

Angiogenesis, the physiological process by which new blood vessels form from pre-existing vessels, is a prerequisite for successful tissue regeneration. Blood vessels are not only conduits for blood flow but are dynamic structures that regulate the microenvironment, providing oxygen and nutrients and removing metabolic waste products [6,7]. Without rapid vascularization, implanted constructs threaten to immediately undergo hypoxia and nutrient deprivation, resulting in cell death and implant failure [7,8]. The control of angiogenesis in engineered constructs is dependent on a balance between pro-angiogenic factors such as Vascular Endothelial Growth Factor (VEGF) and basic fibroblast growth factor (bFGF), which activate and attract and mature the endothelial cells (ECs) to form vascular networks around the scaffolds. Strategies in tissue engineering now include the delivery of growth factors, the use of endothelial progenitor cells (EPCs), and the use of materials that mimic the ECM to encourage vascularization [9].

Vascularization is still one of the major hurdles to be overcome in the field of engineered tissues, despite it being an absolute necessity for the survival of such tissues. The principal limitation is that oxygen and nutrient diffusion are restricted to a distance of about 100–200 micrometers, which is the distance from a capillary [8]. At a farther distance, engineered constructs develop necrotic cores, which are frequently found in large-scale tissue grafts [8,10].

The presently available scaffolds are far from being able to imitate the intricate hierarchical organization of natural vascular networks. On the one hand, synthetic grafts may lack the necessary biological signals for the recruitment of host endothelial cells, while on the other hand, natural scaffolds may have problems of low mechanical strength or high degradation rates, even before the neovascularization process is finished [11]. It is very important and challenging to overcome the time gap between the moment when the scaffold is implanted and the host’s angiogenic reaction, which requires a very sophisticated material design [10].

Vascularization is the stumbling block that has been facing the field of tissue engineering. It is a difficult task to generate living blood vessel patterns that could efficiently supply oxygen and nutrients to thick, metabolically active tissue. Functional vascular networks in living organisms have complex branching, which ensures that cells are within diffusion distance of the blood supply, a feature that is hard to recreate in vitro or after implantation. The diffusion limitations cause steep oxygen gradients, which give rise to ischemia and necrosis in central areas of engineered tissues without perusable vasculature.

For medical use, the fast connection with the host vessels after the implantation is very significant; however, most scaffold materials do not permit sufficient vascular infiltration, particularly when they do not offer intrinsic angiogenic cues. Additionally, the mechanical and biochemical characteristics of cells’ microenvironment also influence their survival and proliferation; hence, biomaterials need to promote pro-angiogenic signaling and extracellular matrix remodeling. Conventional scaffolds are frequently functionally insufficient to yield the desired outcomes, thus postponing the ingrowth of vasculature.

The complexity of vascular biology worsens the problems since angiogenesis involves the tightly regulated interactions between endothelial cells, support cells around them, and a multitude of signaling molecules. Achieving a balance in scaffold design between mechanical strength and permeability for nutrient transport is still a challenge, and researchers are turning to new approaches such as microchannel integration and dynamic culture systems to enhance vascular network formation.

In order to break through these barriers, the research has recently been highly focused on bioinspired polymeric scaffolds that structurally and functionally reproduce the native tissues. Nature serves as a fundamental model for regeneration by the ECM, which is a perpetually changing network of fibers that not only delivers the mechanical framework but also provides essential biochemical and biophysical signals that govern cell adhesion, migration, proliferation, and differentiation.

Bioinspired scaffold concepts have the goal of recreating the cellular microenvironment with advanced polymeric systems, which are architecturally unified by nanofibrous and highly porous constructs to facilitate cell infiltration and integration. On top of that, these scaffolds present the bioactive cues for the endothelial and regenerative cell recruitment, including angiogenic growth factors or functional peptides. Several of the designs are also “smart”, being responsive to environmental factors such as pH or temperature to enable a controllable, on-demand release of the therapeutic agents. Both natural polymers (e.g., collagen and chitosan) as well as synthetic polymers (e.g., polycaprolactone and poly-lactic glycolic acid (PLGA)) are good platforms for such bioinspired systems that permit precisely controlled mechanical properties and degradation kinetics closely matching the needs of the target tissue.

Bioinspired design in tissue engineering is concerned with the development of polymeric scaffolds that mimic the native ECM to enhance tissue regeneration. These scaffolds have hierarchical architecture, suitable mechanical properties, and biochemical signals that promote cellular interactions and vascular integration. Natural or synthetic polymers, such as collagen, chitosan, poly-lactic acid, and polycaprolactone, are used for their biocompatibility and mechanical versatility. The scaffolds are fabricated with adjustable porosity and microchannel networks to facilitate nutrient transport and cellular processes, and materials like electrospinning and 3D bioprinting are used to manufacture structures that resemble capillaries. Furthermore, these scaffolds permit the regulated delivery of essential growth factors and the incorporation of mechanical cues to encourage the formation of vascular networks, thereby facilitating clinical applications of engineered tissues.

There are three main types of tissue engineering vascularization methods: cell-based, material-based, and hybrid. Cell-based techniques, encompassing endothelial cells (ECs), endothelial progenitor cells (EPCs), or mesenchymal stem cells (MSCs), can rapidly establish a biologically relevant vascular network. Co-culture systems can help vessels grow faster. But these methods have problems like low cell survival, immune rejection, high costs, problems with scaling, and problems with regulations. Material-based methods use bioinspired polymeric scaffolds to control the release of angiogenic factors that the host directs for vascularization. These methods might take a while to get going, but they are more reliable, less likely to cause an immune response, and easier to scale up. Hybrid techniques use both types of methods. They help blood vessels grow quickly and give long-lasting structural and biochemical support. Because of this, these methods are the most promising for use in translational applications [8].

Bioinspired scaffolds are used in many different tissues, but their design needs are very different for each type of tissue because of the vascular needs of each type of tissue. Their design needs are very different for each type of tissue because of the specific vascular needs of each type of tissue. Bone tissue needs to be very strong and have large pores (300–500 µm) that connect to each other to support both osteogenesis and vascular invasion. Cardiac tissue needs scaffolds that are both elastic and conductive and can sync up electrical and mechanical activity. Cardiac tissue, on the other hand, needs scaffolds that are both elastic and conductive and can sync up electrical and mechanical activity. Neural tissue engineering focuses on aligned topographic cues and moderate vascularization to promote axonal growth, whereas skin regeneration emphasizes rapid angiogenesis and high porosity for effective wound healing. Topographical cues and moderate vascularization facilitate axonal growth, whereas skin regeneration emphasizes rapid angiogenesis and elevated porosity for effective wound healing [12].

This review covers the recent progress of bioinspired polymeric scaffolds to enhance angiogenesis for tissue regeneration. Angiogenic mechanisms are discussed, along with design strategies such as surface modification, delivery of growth factors, and fabrication techniques, including 3D bioprinting and electrospinning. The functionality of such scaffolds has been discussed in bone, skin, and cardiac regeneration through various models. The translational barriers have also been discussed, and future directions have been suggested, integrating bioreactor platforms, dynamic mechanical stimulation, and computational modeling for enhanced regenerative medicine.

## 2. Fundamentals of Angiogenesis in Tissue Engineering

Angiogenesis is a physiological process whereby new blood vessels are formed out of the existing ones. Angiogenesis of new blood vessels is different than vasculogenesis, which is the creation of vessels by the de novo process of EPCs [7]. Angiogenesis is a very coordinated and multiphase process that is controlled by a strict balance between pro-angiogenic and anti-angiogenic signals in the tissue microenvironment. Angiogenesis in most physiological and pathological situations is triggered by hypoxia induced by tissue injury, which causes the stabilization of Hypoxia-Inducible Factor-1 alpha (HIF-1a). Stabilized HIF-1a, in its turn, induces transcriptional upregulation of major angiogenic mediators, most especially VEGF, hence initiating parent vessel endothelial cell activation [13].

In the parent vessel, a phenotypic switch of the EC lining occurs on stimulation by VEGF, with functional specialization. At the front of the sprout, highly motile so-called tip cells are produced that extend many filopodia to sense and migrate in response to gradients of VEGF. Proliferative so-called stalk cells follow behind, extending the sprout and leading to vessel extension [14]. This sprouting and directed migration phase needs a localized breakdown of the basement membrane and surrounding ECM by matrix metalloproteinases, facilitating the invasion of the endothelial cell into the hypoxic tissue [15]. Endothelial sprouts can then form an anastomosis between them, which is followed by the formation of a lumen that forms functional blood flow in the mature vascular network.

**Figure 1:**
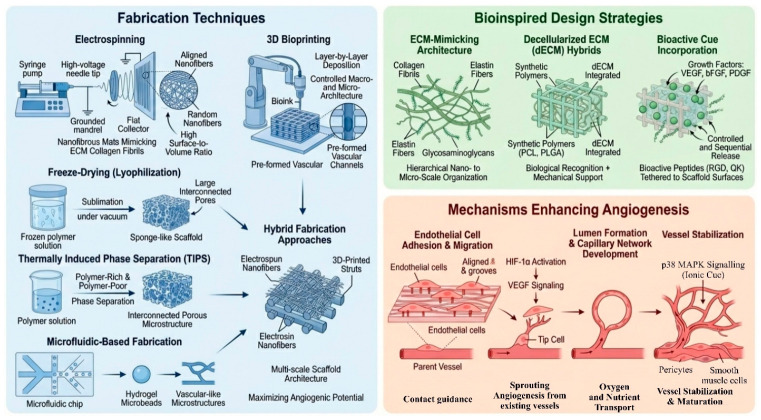
The figure summarizes key fabrication methods for angiogenic scaffolds, and highlights bioinspired designs with the incorporation of bioactive signals such as growth factors and angiogenic peptides. All these strategies boost angiogenesis by promoting endothelial cell adhesion and migration, initiating sprouting angiogenesis, facilitating lumen formation and capillary network development, and lastly, vessel stabilization and maturation. The figure was developed using Nanobanana Pro 3.1 as the image creation software.

Newly issued vessels are structurally immature, hyperpermeable, and unstable at this stage. The last and important step in the angiogenesis process is then vessel stabilization and maturation, which is attained by recruitment and incorporation of mural cells, some of which are pericytes and smooth muscle cells (SMCs). Platelet-Derived Growth Factor (PDGF) and Angiopoietin pathways have a primary role in the regulation of this process, which facilitates the endothelial–mural cell interactions, strengthens vessel walls, controls vessel diameter, and maintains long-term vessel stability [7,16]. The interdependencies among these scaffold requirements, such as biocompatibility, biodegradability, mechanical strength, porosity, and pore architecture—and their collective influence on angiogenesis—are schematically illustrated in Figure 1, which serves as a visual roadmap for the design strategies and fabrication techniques elaborated in the following sections. Upon development, the stabilized vasculature is not only able to restore perfusion to the injured tissue, but it also makes an active biological interface that facilitates regeneration through the delivery of oxygen and nutrients, elimination of metabolic waste, trafficking of immune cells, provision of angiocrine signals to parenchymal cells, and maintenance of perivascular stem cell niches. These well-coordinated molecular and cellular processes are the most important to understand in designing bioinspired scaffolds and tissue engineering approaches that can recreate physiological angiogenesis and attain robust and functional vascularization [7,16].

## 3. Polymeric Scaffolds in Tissue Engineering

The choice of the base material is the most significant step in the design of the scaffolds, and the most commonly used group of polymers is a versatile group because of its universal application. In most cases, these materials are divided into two different groups: natural and synthetic polymers, and each of them has its own benefits and drawbacks that predetermine its use in a particular type of tissue.

Natural polymers (collagen, gelatin, chitosan, and silk fibroin) are of biological origin and are bioactive in nature. Their major strength is that they are molecularly similar to the natural ECM, thereby enabling them to adhere, proliferate, and differentiate better [15]. As an example, decellularized scaffolds based on ECM retain the multifaceted nano-architecture along with biochemical cues required to attract host ECs [15]. However, natural polymers have some major weaknesses, such as batch-to-batch variations, uncontrolled and rapid degradation rates, and low mechanical performance, which restrict their application in load-bearing applications such as bone tissue engineering [17,18]. On the other hand, synthetic polymers, such as poly-lactic acid (PLA), poly glycolic acid (PGA), and polycaprolactone (PCL), provide an accurate control of mechanical strength, degradation kinetics and fabrics reproducibility [18,19]. These materials are able to be molded into complicated shapes with high mechanical fidelity. However, their biggest weakness is a lack of sites of biological recognition; their hydrophobic surfaces can frequently cause subpar cell attachment and even cause a foreign body response [20].

Increasingly, scaffold design has focused on hybrid polymeric materials that incorporate natural and synthetic phases within a single system, as a means of overcoming the limitations of monolithic systems. This approach is justified by comparative studies that demonstrate hybrid systems consistently outperform mono-material systems in both biological and mechanical terms. For instance, collagen–PCL hybrid scaffolds have demonstrated enhanced mechanical performance and stability over pure collagen scaffolds, while also facilitating greater endothelial cell adhesion and cell spreading than pure PCL scaffolds as a result of the inclusion of native cell-binding domains [21,22]. Similarly, PLGA–gelatin hybrid scaffolds have been shown to increase cell viability, wettability, and vascular infiltrate compared with PLGA scaffolds, suggesting that the addition of a natural phase can directly enhance early angiogenic events while maintaining structural integrity [21,22].

This combination of improved performance results from the distinct functions of the two phases: the synthetic phase provides structural integrity, shape retention, and controllable degradation, while the natural phase provides biological recognition, hydrophilicity, and enhanced communication between cells and the matrix [21,22]. Additional studies also demonstrate that hybrid scaffolds exhibit more balanced degradation profiles than natural polymers and more optimal endothelial cell attachment than unmodified synthetic polymers, thus enhancing scaffold survival and integration [21,22]. In the context of angiogenesis-based tissue engineering, this is a critical consideration, since vascularization necessitates early endothelial cell recruitment and sufficient long-term support to sustain tissue growth and development. As a result, hybrid polymer systems are now recognized as one of the most successful approaches to scaffold design for achieving mechanical stability, biological functionality, and long-term angiogenic response [21,22].

Polymer–ceramic composite scaffolds are a critical type of multifunctional biomaterials that aim to address the mechanical and biological requirements of vascularized bone and osteochondral tissues. These scaffolds combine the processing flexibility, elasticity, and adjustable degradation of polymers such as polycaprolactone (PCL), poly-lactic acid (PLA), poly-lactic-co-glycolic acid (PLGA), collagen, and gelatin with the biomechanics, osteo-conductivity, and angiogenesis-promoting properties of bioactive ceramics such as hydroxyapatite (HA), β-tricalcium phosphate (β-TCP), and bioactive glass (BG) [23]. This combination overcomes the deficiencies of polymers (poor bioactivity) and ceramics (brittleness), yielding scaffolds with combined and tunable properties. Polymer/ceramic composites consistently perform better than pure polymers by providing improved mechanical properties, surface roughness, and osteoblast adhesion, and by stimulating endothelial recruitment via calcium-, phosphate-, and silicate-induced signaling [23]. Ion release from the ceramic components also promotes endothelial cell migration and VEGF production, leading to simultaneous mineralization and angiogenesis. Micro- and macro-fabrication technologies, such as additive manufacturing, provide highly controlled ceramic distribution and hierarchical, patient-specific scaffold designs with enhanced mechanical properties and biological responses [24,25]. In all, these composites provide bio-instructional rather than structural support and are promising for new-generation scaffolds with both mechanical and vascularization capabilities [23,24,25].

### 3.1. Key Properties Required for Scaffolds: Biocompatibility, Biodegradability, Mechanical Strength, Porosity

The design of polymeric scaffolds to promote good angiogenesis and long-term tissue integration requires multiple physicochemical and biological criteria. While these characteristics are interrelated, their significance is unequal and should be ranked according to their direct impact on angiogenesis and tissue integration. When designing scaffolds to induce angiogenesis, the first priority is biocompatibility, as an otherwise structurally suitable scaffold can still fail if it leads to chronic inflammation, thrombosis, and/or encapsulation post-implantation [18,22].

*Biocompatibility* is an essential characteristic of scaffold design, but it cannot be considered a binary attribute. Rather, it is a complex, quantitative, and dose- and site-dependent property that depends on degradation processes, conditions of exposure, and scaffold structure. Therefore, a comprehensive assessment must consider a range of toxicity-related factors that affect cell response and function. For a polymeric scaffold to successfully induce tissue regeneration and vascularization, it must satisfy a stringent set of physicochemical and biological criteria. The “tissue engineering triad” relies heavily on the scaffold acting as a temporary template that mimics the native cellular environment [19]. Biocompatibility is the paramount requirement; the material must integrate with the host tissue without eliciting a severe immune rejection or chronic inflammatory response that would lead to fibrous encapsulation, effectively blocking angiogenesis [26]. The scaffold surface should support cell attachment and migration, often requiring specific nano-topography or chemical functionalization [16]. Polymers like PLA [27], PCL [28], and PLGA [29] are well-established for their safety and regulatory approval.

*Biodegradability* is also very important. The scaffold is not created as a permanent aid, and it should be such that it decays at the same rate as new tissue grows. In the case of excessive degradation, the construct can fail mechanically; in the case of too little, it can slow down the integration of tissues. Notably, the byproducts of the degradation should be non-toxic and should be readily metabolized or excreted by the body [30]. The metabolite at the end of the degradation process must be non-toxic, e.g., lactic or glycolic acid, which can be biologically processed or eliminated. When there is inadequate scaffold degradation in relation to tissue maturation, mechanical failure or failure of full tissue regeneration may occur.

The dose–response relationship of degradation products and leachables is a major factor affecting biocompatibility. Synthetic biodegradable polymers (e.g., PLA, PGA, PLGA) degrade by hydrolysis to release acidic degradation products (e.g., lactic and glycolic acid) that can accumulate locally and drop the pH level to affect cell viability, unless they are well-buffered or released [26]. This effect is a dose-dependent process, with materials showing acceptable non-toxicity at lower concentrations, but becoming toxic as their degradation products accumulate. For example, cytocompatibility standards set by ISO 10993-5 [31] require a cell viability of ≥70%, but the same material may appear non-toxic at dilute concentrations and toxic at higher concentrations. Likewise, degradation toxicity is time-sensitive, as some polymers (e.g., PGA) become highly toxic after prolonged exposure, while slower-degrading materials (e.g., PCL) remain less toxic. These results highlight the importance of controlling the degradation rate and concentration of the released species [26].

The conditions of exposure also affect the toxicity of scaffolds. Factors such as medium/scaffold volume ratio, exposure time, and local volume affect the concentration (or dilution) of degradation products. ISO 10993-12 [32] requires a standard extraction ratio, as different ratios can give misleading results: a large ratio may dilute toxic byproducts, resulting in a false-negative result, while a small ratio may overestimate toxicity. Likewise, the local environment (pH, medium, mechanical stimuli) is important. Acidic degradation can reduce physiological pH from ~7.4 to <6.0, causing inflammation and preventing new blood vessel formation, while proteins in biological media can neutralize or bind toxic byproducts, decreasing their toxicity. Perfusion or mechanical stimuli can increase mass transport and wash away degradation byproducts, which typically leads to better biocompatibility than static assays [33,34].

Scaffold architecture adds another layer of complexity as it affects both cell–scaffold interactions and the overall “dose” of material. Highly porous or nanofibrous scaffolds have a high surface area-to-volume ratio, which increases the rate of protein adsorption and cell attachment but also promotes the degradation and release of degradation products [33]. For instance, electrospun nanofibrous scaffolds can have 100–500 times more surface area than their bulk counterparts, which could result in higher concentrations of degradation byproducts. On the other hand, dense or poorly connected scaffolds can limit diffusion, leading to local pockets of acidic or pro-inflammatory byproducts. Pore size and interconnections are critical: large, interconnected pores enable fluid convection and byproduct transport, while small or isolated pores can accumulate degradation products and generate cytotoxic pockets [33,34]. Additionally, surface properties such as roughness, nano-topography, and chemical modifications modulate cellular interactions by regulating focal adhesion assembly, cytoskeletal tension, and endothelial functions [15,35]. But morphological degradation may also create particulate debris or highly energetic nanoparticles that can trigger macrophages and cause oxidative stress or inflammatory reactions [22]. Thus, biocompatibility should be considered in terms of the dynamic interactions between chemistry, degradation, exposure, and scaffold structure. Considering these factors in scaffold design allows for better in vivo performance prediction and is critical for the safe, effective, and translatable design of angiogenic biomaterials [15,22,26,33,36].

The second most critical property is pores (porosity and pore interconnectivity), which regulate oxygen and nutrient supply, vascular invasion, and capillary formation directly within the scaffold. Biodegradability is the next most important parameter and must be timed to match the rate of tissue regeneration so the scaffold acts only as a temporary scaffold to support regeneration, without hindering matrix remodeling or vascular maturation [26]. The fourth priority is mechanical properties and is highly tissue-specific; while important for scaffold stability and mechano-transduction, some tissues, such as soft tissues, do not require as much mechanical support as others, such as bone [27,28]. The fifth priority is surface functionality (topography and chemical properties) because it is a secondary factor that amplifies cell attachment, endothelial migration, and bioactive signaling, but it is not a major driver of scaffold performance [15].

Interconnectivity and nanoscale porosity are key metrics of scaffold performance, along with bulk porosity and estimated pore size, and should be considered fundamental design parameters for angiogenic scaffolds. Although total porosity represents the total volume fraction of void spaces available for tissue ingrowth, the effectiveness of these voids is determined by pore interconnectivity. 

It also has limited cell mobility and poor vascular integration, etc., despite total porosity being high [7,30]. Conversely, well-connected pore networks allow continuous transport pathways for nutrients, gases, and waste products, facilitating uniform tissue regeneration and long-term vascularization within a scaffold.

Connectivity is a key element of angiogenesis. To establish a continuous path between the peripheral and central portions of a scaffold for the growth of new blood vessels, capillaries need to create a path from their origin (the scaffold) to the surrounding tissue. Sufficiently interconnected structures will maximally increase the hydraulic conductivity (i.e., the ability to conduct fluid), minimize the likelihood of forming areas with low oxygen availability (“dead zones”), and facilitate an equal distribution of metabolites, which can improve the viability of cells and increase vascular connectivity [30]. In contrast, poorly interconnected scaffolds are likely to result in ischemic (i.e., areas with low oxygen) areas, and will have inconsistent patterns of blood vessel development, even if they exhibit adequate overall porosity [7].

On smaller scales, nanoscale porosity provides an additional dimension of control. Nanoporous features enhance the surface area-to-volume ratio, and promote protein adsorption, growth factor retention, and integrin binding for cell adhesion at the cell–material interface [7,15,33]. This can also increase microscale fluid flow, such as capillary transport of water and short-distance diffusion of oxygen, which is critical in dense matrices such as hydrogels or nanofibrous scaffolds, where long-range transport may be hindered [33,35]. And finally, nanoscale porosity affects matrix swelling and ion transport, which in turn affects endothelial cell function and tissue regeneration. Overall, for improved scaffold function, a multiscale pore structure is required to accommodate cell invasion and vascular ingrowth (interconnected macro- and microporosity) as well as local biochemical and fluid transport (nanoscale porosity). This hierarchical structure more closely resembles the native extracellular matrix and is critical for mass transport, oxygen delivery, and vascularization in engineered tissues [7,35].

The most significant aspects of scaffold design within the context of vascular tissue engineering can be prioritized as follows: biocompatibility, pore architecture, biodegradability, mechanical properties, and surface functionality (Table 1). 

Biomechanical properties of scaffolds are very specific for different tissues and should closely resemble the mechanical properties of the tissue of implantation; thus, there is “no one size fits all” approach. In weight-bearing tissues like bone, stiff and strong scaffolds are imperative (50–150 MPa compressive strength) to support physiological loads, provide structural integrity, and support bone regeneration [28]. For soft tissues, more elastomeric materials are required. For instance, skin scaffolds should be able to stretch and contract to allow flexibility, with a reported tensile strength of 2–30 MPa and low elastic modulus (0.4–0.8 MPa). Other dynamic tissues, such as cardiac muscle, require soft elastomeric scaffolds that can withstand cyclic contraction–relaxation; stiff scaffolds in such systems can compromise cardiomyocyte function and electromechanical coupling [28,29]. The brain is an extremely delicate environment where ultra-soft scaffolds with low stiffness are needed to avoid glial activation and axonal regeneration inhibition. In summary, scaffold design should not only avoid mechanical failure but also provide biomechanical compatibility with the host tissue to allow for appropriate cellular functions, vascularization, and transmission of mechanical stimuli, which are necessary for maintaining cell phenotype and differentiation, as shown in Table 2 [28,29].

The vascularization is dependent upon porosity and pore architecture. A network of porous structure (usually >90% porosity) is needed to enable the diffusion of oxygen and nutrients, elimination of wastes, and most importantly, physical infiltration of ECs to create capillary networks. The pore size should be optimized (e.g., 200–400 µm in bone) to avoid hypoxic zones whilst preserving structural integrity [8,12]. The porosity of the scaffold determines the diffusion of nutrients, oxygen, and vascular ingrowth. The size of the optimum pore size is between 50 and 150 µm in skin, 150–300 µm in cartilage, and 300–500 µm in bone regeneration. These interconnectivities of the pores mediate cell migration and neovascularization, and these are vital in tissue integration and long-term survival.

The efficiency of scaffolds is highly dependent on their porosity and pore architecture, which affect gas and nutrient transport, waste elimination, cellular migration, and the ingrowth of blood vessels. But the ideal porosity differs among tissues and depends on the physiological and biomechanical demands of the tissue of interest [30]. In general, a highly porous and interconnected network (porosity > 90%) is required for efficient mass transport and ongoing endothelial migration to allow efficient and even neovascularization [30]. However, optimal porosity is not enough; pore size is critical in determining the nature and extent of cell infiltration.

For bone tissue engineering applications, larger pores (300–500 µm) are desirable for weight-bearing tissues, as they allow ample diffusion. For cartilage regeneration, mid-sized pores (150–300 µm) are desirable, as they allow sufficient diffusion of nutrients while not compromising the density of the matrix and supporting chondrocyte aggregation and extracellular matrix production in a non-vascular environment [30]. However, smaller pore sizes (50–150 µm) are preferred for soft tissues such as skin, as they facilitate fibroblast attachment, rapid re-epithelialization, and the formation of fine capillaries because of the increased surface area-to-volume ratio [7].

Critically, pore interconnectivity is just as important as pore size, as closed pores limit tissue infiltration and vascularization, despite higher overall porosity [30]. Although increased porosity enhances mass transfer and vascularization, it may also compromise the mechanical stability and cause failure of the scaffold, especially in load-bearing tissues. Hence, scaffold design optimization should take into account the balance between pore size, interconnectivity, and total porosity to provide a sufficient mechanical integrity and vascular network, instead of considering the porosity alone [30].

### 3.2. Methods of Fabrication

The development of technologies in manufacturing has made it possible to develop scaffolds whose architecture can be controlled with great precision, which means that researchers can recreate the hierarchical structure of native tissues.

Electrospinning is an eminent method of producing fibrous scaffolds that are very similar to the nanoscale of the original ECM. This technique is used to manufacture non-woven nanofiber mats of high surface-to-volume ratios by exposing a solution of polymer to a high-voltage electric field [43]. Such nanofibrous networks are especially effective in endothelial cell migration and can be angiogenic factor functionalized. Adjustments in the alignment of the fiber enable the process of making anisotropic structures that are optimal in nerve, tendon, and vascular tissue engineering. The bioactive molecules which are incorporated by means of electrospinning include growth factors and nanoparticles and are utilized in controlled release to enhance angiogenesis and osteogenesis. Nevertheless, traditional electrospun mats tend to be accompanied with small pore sizes that might inhibit cellular infiltration at the surface [44,45].

Electro-spraying is an electrohydrodynamic technique similar to electrospinning and is increasingly being used in tissue engineering to produce particles and deliver bioactive agents [33,46,47]. Electrospinning is used to fabricate continuous ECM-like fibrous scaffolds, whereas electro-spraying involves less concentrated polymer solutions that are used to fabricate homogenous micro- and nanoparticles [33]. In the design of scaffolds, electro-spraying can be used to produce polymeric particles for surface coating, microspheres and encapsulation of sensitive cargo such as growth factors and genetic material under mild processing conditions [33]. These particles have greater size control, encapsulation efficiency and presentation of active cues than traditional methods. So, electrospray systems are commonly used to deliver angiogenesis-promoting factors, such as VEGF, PDGF, SDF-1 and bFGF, which can be delivered in a sustained and spatially confined manner without premature degradation [46,47].

3D bioprinting has transformed the fabrication of scaffolds by providing a means of control of the macro- and microarchitecture. In this method of additive manufacturing, layer-by-layer deposition of so-called bioinks (polymers typically loaded with cells) can be used to produce patient-specific constructs guided by medical imaging data [48]. 3D printing enables the production of scaffold architecture, pore size, and geometry with high spatial resolution. Such methods as fused deposition modeling (FDM), semi-solid extrusion (SSE) 3D printing, and stereolithography (SLA) are employed to fabricate patient-specific scaffold structures using polymers, hydrogels or composite materials, depending on the specific printing modality. Most importantly, 3D bioprinting allows the formation of pre-assembled vascular channels in the scaffold to bypass the diffusion barrier by establishing an intrinsic plumbing system to provide immediate perfusion [49,50]. Despite these advantages, challenges remain in maintaining high cell viability during printing, achieving sufficient mechanical strength, and ensuring long-term vascular stability following implantation. This approach demonstrates strong potential to make personalized implants that closely fit the patient anatomy, which will be integrated and functional perfectly with host tissue. Moreover, recent developments in 4D bioprinting currently permit dynamic responses of scaffolds to physiological conditions (e.g., pH or temperature) to enhance adaptivity and in situ maturation [51]. To complement the macroscale precision of printing-based approaches, techniques such as freeze-drying (lyophilization) and thermally induced phase separation (TIPS) are often integrated to generate extremely porous sponge-like architectures required for cell infiltration. Additionally, microfluidic-based approaches have also been increasingly used to develop hydrogel microbeads or fine details of vascular structure, which offers a high level of control of the microenvironment [14,52]. The selection of fabrication techniques is largely dictated by the requirements of the target tissue, and recent strategies increasingly favor hybrid approaches to scaffold fabrication—e.g., electrospun fibers with 3D-printed struts—to synergistically combine nanoscale biomimicry with macroscale structural support and make the most of the angiogenic potential [53]. Such hybrid and multiscale fabrication strategies have gained considerable attention for their ability to produce scaffolds that simultaneously achieve architectural precision, biological relevance, and functional complexity, thereby bridging the interface between biology and engineering.

## 4. Bioinspired Design Strategies

### 4.1. Inspiration from Nature: Extracellular Matrix—Mimicking Designs and Materials

The fundamental premise of bioinspired design is that the ECM is not merely a passive structural support but an instructive microenvironment that dictates cellular fate. Consequently, the “gold standard” for scaffold fabrication is to replicate the native ECM’s physicochemical properties [54]. The native ECM is a dynamic, fibrous composite consisting of proteins (collagen, elastin) and polysaccharides (glycosaminoglycans) organized into a hierarchical nanofibrous mesh. This architecture provides critical contact guidance for endothelial cells, directing the sprouting of new capillaries [15,55].

Fibrous scaffolds, which resemble the architecture of the extracellular matrix (ECM), provide topographical and mechanical signals that shape how cells align, move, and differentiate by mimicking both an ECM and the underlying ECM. Unlike traditional non-bioinspired scaffolds, such as hydrogels or sponges, that allow only for passive transport of nutrients to a randomly infiltrating population of cells, fibrous scaffolds enable precise spatial alignment of the cells so they can form an organized, efficient vascular network. As a result, electrospun nanofibrous scaffolds (50–1000 nm) exhibit high surface area-to-volume ratios, allowing for enhanced adsorption of proteins and rapid attachments of endothelial cells early in their development, which in turn greatly accelerates directional endothelial sprouting and leads to the development of well-organized, mature vascular networks with more blood flow than would be possible with traditional scaffolds [43]. In addition to their structural characteristics, ECM-like scaffolds also create an extracellular microenvironment similar to that of native tissues, thereby providing ECs with biomimetic signals that promote EC adhesion, EC migration, and EC invasion into the surrounding extracellular matrix (e.g., via use of biomimetic signals) [14,48]. Their primary strength lies in facilitating long-term biological integration and sustained cell-instructive signaling that closely resembles natural tissue remodeling [49]. However, because they rely largely on host-driven vascular infiltration, these scaffolds often exhibit slower initial angiogenic responses, particularly in large or ischemic defects when used alone [10].

Growth factor delivered approaches (e.g., VEGF, bFGF, PDGF) can provide quick endothelial activation and early angiogenesis; however, they have limitations, such as short half-life, burst release, and no long-term control [53,54]. Cell-based approaches form a primitive vascular network in the early stages of the host’s body, so there is more direct access to creating vascular structures. However, challenges still exist, such as cell survival, scalability, and complex regulations related to the use of cells to develop vascular structures [7,38].

### 4.2. Incorporation of Bioactive Cues (e.g., Growth Factors, Peptides)

While structural mimicry is essential, physical architecture alone is often insufficient to trigger rapid angiogenesis in large defects. To address this, bioinspired scaffolds are frequently functionalized with bioactive cues, specifically angiogenic growth factors and functional peptides [56].

Growth factors such as VEGF, bFGF, and PDGF are the most potent drivers of angiogenesis. However, the direct injection of these factors often leads to rapid clearance or off-target effects. Therefore, bioinspired scaffolds utilize controlled release systems—such as encapsulation within hydrogels or binding to heparinized surfaces—to mimic the sustained, localized availability of factors found in the native niche [56,57]. For example, dual-delivery systems that release VEGF (for vessel initiation) followed by PDGF (for vessel maturation) have shown superior stability in formed vascular networks compared to single-factor delivery [57]. Recognizing the high cost and instability of whole proteins, research has shifted toward short bioactive peptides. These peptides, such as the RGD (arginine–glycine–aspartic acid) sequence or QK peptide (a VEGF mimic), are more stable and can be covalently bonded to the scaffold backbone [58]. Self-assembling peptide hydrogels can form nanofibrous structures that display a high density of these bioactive epitopes, effectively “fishing” for ECs from the surrounding tissue [59]. Recent work has also highlighted the role of immune-modulating peptides which, when loaded into implants, can shift the host response from inflammation to regeneration, indirectly fostering a pro-angiogenic environment [60].

### 4.3. Topographical and Chemical Modifications to Enhance Cell Attachment and Behavior

Cell behavior is profoundly influenced by the surface properties of the substrate. Bioinspired strategies exploit this by engineering specific topographical and chemical modifications to guide EC attachment, migration, and alignment [16].

Topographical modifications involve creating surface patterns at the micro- and nanoscale. It is well-established that ECs align and migrate along grooves and ridges, a phenomenon known as contact guidance. Bioinspired surface topographies, such as nanogrooves or pillar arrays, have been shown to accelerate EC migration speeds and promote the formation of capillary-like structures in vitro even in the absence of growth factors [16]. Advanced techniques like laser texturing are used to imprint these patterns onto polymer surfaces, significantly enhancing the scaffold’s integration with the host vascular network [36].

Chemical modifications alter the surface energy and charge to improve hydrophilicity, as most synthetic polymers are hydrophobic and repel cell attachment. Plasma treatment is a standard method to introduce oxygen-containing functional groups, increasing surface wettability [61]. More advanced chemical strategies involve the “mussel-inspired” deposition of polydopamine (PDA). PDA coatings not only improve cell adhesion universally but also serve as a versatile platform for secondary functionalization, allowing for the easy immobilization of angiogenic factors or metallic ions [35]. Furthermore, incorporating specific chemical elements, such as magnesium or silicon ions, into the scaffold surface has been shown to develop a localized biochemical environment that stimulates angiogenesis via the activation of specific intracellular pathways (e.g., HIF-1α or p38 MAPK) [62,63]. Figure 1 provides a comprehensive schematic overview of the key fabrication techniques, bioinspired design strategies, and the sequential cellular mechanisms that collectively enhance angiogenesis. As shown in the figure, the process begins with scaffold fabrication methods (electrospinning, 3D bioprinting, freeze-drying, etc.) that generate specific topographical and biochemical cues. These cues then promote endothelial cell adhesion and migration, initiate sprouting angiogenesis, facilitate lumen formation and capillary network development, and finally support vessel stabilization and maturation. The reader is encouraged to consult Figure 1 while reviewing the following sections, as it visually integrates the multi-step angiogenic cascade described throughout this manuscript.

## 5. Mechanistic Insight on Improvement in Angiogenesis Through Bioinspired Polymeric Scaffolds

Angiogenesis is essential for the successful retention of engineered tissue over time. Bioinspired polymeric scaffolding can enhance the overall function of these engineered tissues by promoting angiogenesis. Bioinspired scaffolds have been developed to help replicate the ECM’s attributes (including dynamic cellular interactions) via bioengineering approaches that alter both surface chemistry, micro/nano-morphology, biochemical delivery, and cellular integration methods [19].

### 5.1. Surface Chemistry and Topography Promoting Endothelial Cell Migration and Vascularization

Surface chemistry is a key and quantitatively adjustable factor in scaffold-mediated angiogenesis, as it controls protein adsorption, endothelial cell adhesion, proliferation, and network formation. Unmodified synthetic polymers (e.g., PCL, PLGA) are hydrophobic (water contact angles > 110°) [64] and exhibit low protein adsorption and cell adhesion. Surface treatments, such as plasma activation, increase the density of oxygen-containing functional groups, leading to a marked increase in hydrophilicity (contact angles < 60°) and initial endothelial adhesion by ~1.5–2-fold due to increased adsorption of adhesion-promoting proteins [52,57]. More sophisticated strategies, like polydopamine (PDA) coating, lower contact angles further to highly hydrophilic values (<40°) and lead to a 2–3-fold increase in endothelial attachment, growth, and spreading, as well as being able to provide functional groups for secondary immobilization of angiogenic ligands [58]. These surface modifications result in increased focal adhesion, cytoskeleton formation, and accelerated formation of an endothelial monolayer [15,58]. This can boost the angiogenic response by covalently attaching bioactive ligands, angiogenic peptides, or creating heparin-like surfaces. These changes push endothelial cells to migrate between 30 and 50% more and ramp up capillary tube formation [55,62]. If bioactive cations added like magnesium or silicates, they kickstart angiogenic signaling pathways—think p38 MAPK and focal adhesion kinase—and get endothelial cells multiplying faster. At the same time, they raise levels of pro-angiogenic genes like Vascular Endothelial Growth Factor and Hypoxia-Inducible Factor-1 [50]. Chemical tweaks help too. For example, adding PMEP to PLGA scaffolds cuts pro-inflammatory cytokines like IL-6 by more than half and bumps up angiogenic factors like VEGF to three times the baseline. All this develops a much better biochemical environment for angiogenesis [65]. At the nano-scale, the surface roughness of biomaterials has been shown to influence endothelial function, with greater alignment, migration, and initiation of lumen formation occurring on nano-rough (50–200 nm) surfaces [36]. In conclusion, these examples illustrate that surface chemistry is not only a qualitative attribute but also a quantifiable parameter that governs endothelial cell behavior, has an anti-inflammatory effect, and enhances overall angiogenic response [36,50,55,62,65].

### 5.2. Controlled Delivery of Angiogenic Factors

One of the major mechanisms through which bioinspired polymeric scaffolds promote angiogenesis is the spatiotemporally controlled delivery of angiogenic molecules. Angiogenesis is a process regulated by a cascade of growth factors such as VEGF, bFGF, and PDGF that, in combination, stimulate ECs’ proliferation, migration, and vessel maturation. Localized sustained release of these factors can be achieved by incorporating them into polymer matrices. In natural wound healing, the release of growth factors occurs in a very specific cascade; bioinspired scaffolds work to recapitulate this dynamic profile rather than just delivering a simple bolus, which gets degraded very fast [56].

Scaffolds are materials that help the delivery of vulnerable proteins, such as VEGF and bFGF, as they protect them from enzymatic degradation. Highly sophisticated dual-delivery systems are structured to follow the biological angiogenic sequence: they let out VEGF rapidly to start vessel sprouting, and then PDGF’s release is prolonged to recruit pericytes for vessel maturation and stabilization [66]. This sequential release is very important because, usually, VEGF only leads to leaky, immature vessels [56].

Apart from this, some newly developed methods support the delivery of bioactive ions (e.g., Copper, Cobalt, Zinc) that are released from ceramic–polymer composites. These ions provide a more affordable and stable alternative to growth factors. For instance, Cobalt ions can act as hypoxia chemically and thus can induce the host angiogenic response intrinsically [13,62]. On the other hand, “smart” hydrogels can release these factors when they detect changes in their surrounding environment, e.g., the lower pH that is characteristic of the ischemic tissue, thus allowing the therapy to be delivered at the exact time and place where it is needed [67,68].

By embedding such molecules in polymer matrices, scaffolds are able to maintain the release of these molecules locally, thus creating concentration gradients that are similar to those in physiological angiogenic niches. Polymeric materials such as PLGA, polyethylene glycol diacrylate (PEGDA), and chitosan-based hydrogels are especially suitable for controlled release purposes as their degradation can be controlled by varying the polymer composition and the extent of crosslinking [69]. For instance, VEGF encapsulated in PLGA microspheres that are then implanted in collagen scaffolds provides a slow-release profile which encourages both the early branching of vessels and the later stabilization. At the same time, heparin-functionalized hydrogels not only stabilize the growth factors but also keep them active, thus leading to ample formation of endothelial networks under both in vitro and in vivo conditions [38]. On top of that, smart scaffolds that are just emerging can also dynamically respond to environmental inputs such as pH, temperature, or the presence of reactive oxygen species. They can thus deliver angiogenic stimuli only when and where they are needed, in response to local tissue conditions. Such biomimetic delivery significantly lessens the possibility of systemic side effects and gives more precise control of the vasculature during tissue regeneration [70].

### 5.3. Integration of Vascular Cells or Pre-Vascularization Strategies

In order to get past the “diffusion limit”, which is the reason for central necrosis in large constructs, bioinspired strategies are thus progressively shifting from passive recruitment to active pre-vascularization. This refers to the development of a preliminary vascular network within the scaffold before or at the time of implantation [8]. Commonly, endothelial cells, EPCs, and pericytes are aimed to be merged into bioinspired polymeric scaffolds so as to start in situ vessel formation. These cells become one unit and form capillary-like structures inside the scaffold, which, later, can connect with the host vasculature after implantation, therefore resulting in faster perfusion and longer tissue survival [71].

One of the most efficient techniques is cell co-culture, where ECs are paired with supportive cells, like fibroblasts, mesenchymal stem cells (MSCs), or pericytes, which can even further improve vascular maturation by offering paracrine signaling and stabilizing perivascular support. The supporting cells not only bring the essential ECM proteins but also secrete paracrine factors that provide both structural and functional support to the newly formed capillaries. Experiments reveal that electrospun scaffolds pre-seeded with this combination can establish functional anastomoses with the host circulation much quicker than monocultures [44]. For instance, hybrid PCL–collagen scaffolds incorporating EC–MSC co-cultures exhibited significantly enhanced vessel density and lumen formation relative to monoculture systems [72]. Furthermore, microfluidic and 3D bioprinting technologies are now enabling the production of pre-patterned microchannels that closely resemble native capillary architectures, thus speeding up the perfusion process post-implantation [73].

Spatiotemporally regulated delivery of angiogenic molecules is another significant process that the bioinspired polymeric scaffolds stimulate angiogenesis. A cascade of growth factors, including VEGF, bFGF, and PDGF, is involved in the regulation of angiogenesis and acts synergistically to induce the ECs’ proliferation, migration, and vessel maturation [74]. Scaffolds can maintain localized delivery by loading these molecules within polymer matrices. Growth factors are emitted in a highly regulated cascade in physiological wound healing; bioinspired scaffolds are expected to reproduce this dynamic profile instead of delivering a simple bolus injection that decays quickly [56].

Scaffolds are reservoirs that can safeguard labile proteins—e.g., VEGF and bFGF. The modern dual-delivery systems are programmed to recreate the angiogenic cascade: a rapid burst of VEGF is used to induce vessel sprouting, and sustained release of PDGF is done to bring pericytes to maturation and stabilize the vessel [66]. This stage-by-stage discharge is essential, and VEGF in isolation frequently causes leaky immature vessels [56]. In addition to proteins, there are recent approaches to the delivery of bioactive ions (e.g., Copper, Cobalt, Zinc) that are discharged by ceramic–polymer composites. The ions are cheaper and more stable compared to growth factors. As a case study, the Cobalt ions may be used in chemo-mimicking hypoxia and inducing the endogenous angiogenic repair in the host [13,62]. Moreover, hydrogels that are intelligent can detect these factors and release them on environmental cues, e.g., the reduced pH of ischemic tissue, so that therapy is actually delivered only when and where it is most required [67,68].

With these molecules fitted in polymer matrices, scaffolds have been able to maintain their localized release, thus forming concentration gradients that recapitulate physiological angiogenic niches. Hydrogels made of polymeric materials, including PLGA, PEGDA, and hydrogels made of chitosan, have a unique benefit in terms of controlled release since the rate at which they degrade can be engineered by changing the composition of the polymer and the density of the crosslinking linkages [69]. As an illustration, the role of the VEGF-loaded PLGA microspheres incorporated into collagen scaffolds to support a longer release profile that facilitates early sprouting of vessels as well as long-term stabilization. On the same note, heparin-functionalized hydrogel enhances the stability of growth factors and their bioactivity, resulting in a strong subsequent endothelial network formation in vitro and in vivo [38].

Smart scaffolds are also emerging that are dynamically responsive to environmental factors like pH, temperature, or reactive oxygen species, which give out angiogenic cues only when required based on the local tissue conditions. The biomimetic delivery method will minimize the risk of invasive side effects and enable better control of the vessel during tissue regeneration [70]. Alternatively, 3D bioprinting enables one to make scaffolds with intrinsic, perfusable microchannels coated with ECs. These direct-printed or sacrificial channels are a direct plumbing system, enabling culture media (in vitro) or blood (in vivo) to flow into the construct in real time [49,75]. This biomimetic engineering guarantees that deep tissues are supplied with oxygen instantly after implantation, overcoming the critical period that occurs between the time the capillaries of the host infiltrate the implant [14,76]. In vivo pre-vascularization may also be realized by placing scaffolds subcutaneously and allowing them to be ingrown by host vessels and then transplanting them to the target site. It has already been applied to bone and cardiac tissue engineering, where rapid revascularization is crucial to graft survival in this form of two-stage implantation [70].

Bioinspired polymeric scaffolds facilitate angiogenesis in a synergistic manner in physical, chemical, and biological conditions. Endothelial behavior is regulated by surface biofunctionalization and nano-topography, and sustained angiogenic signaling is ensured by controlled delivery systems. At the same time, the incorporation of vascular cells or pre-vascularization strategies promotes faster functional and perfusion incorporation. The combination of these mechanisms form a complex strategy of vascularized tissue engineering, which allows one to develop living and functional tissues with long-term survival and regeneration.

## 6. Bioinspired Polymeric Scaffolds

Bioinspired polymeric scaffolds have been designed to mimic the structural hierarchy, biochemical, and dynamic responsiveness of the natural ECM. They may be in general divided into hydrogels, fibrous scaffolds, multi-layered or composite scaffolds, and smart responsive scaffolds, depending on their physical shape and functional goals. Both types have distinct mechanical and biological benefits in the stimulation of cell growth, angiogenesis, and tissue formation in a wide range of biomedical models.

### 6.1. Hydrogels (Natural/Synthetic and Composites)

Hydrogels are considered one of the most versatile types of bioinspired scaffolds because they consist of a lot of water and are physicochemically comparable to the original soft tissue ECM [77]. These cross-linked, three-dimensional polymer networks offer an ideal diffusive microenvironment to oxygen and nutrients, which is essential in supporting the early development phases of angiogenesis [70].

Natural hydrogels have the advantage of being bioactive; a number of them are based on polymers such as collagen, gelatin, fibrin, and chitosan. They include inherent cell-binding motifs (e.g., RGD motifs) which predisposes endothelial cell attachment and migration naturally [70]. They have, however, poor clinical usefulness due to high rates of degradation and poor mechanical characteristics. Synthetic hydrogels (e.g., PEG, polyvinyl acetate, polyacrylamide), on the other hand, have better mechanical stability and degradation rate and can be functionalized but have no biological cues [78].

Composite hydrogels of natural and synthetic polymers have become multifunctional biomaterials to overcome individual limitations. Researchers can design scaffolds having natural scaffolds reinforced with synthetic networks or nanoparticles, which include inorganic scaffolds (such as hydroxyapatite), to gain biological recognition and structural integrity [79]. Indicatively, PEG–gelatin or PCL–alginate hybrids augment bioactivity and mechanical strength. Also, a nanoparticle or bioactive ceramic (e.g., hydroxyapatite, silica) incorporation into hydrogel matrices enhances the angiogenic factor retention and controlled release. Recent progress in 3D bioprinting of hydrogel-based inks has enabled the creation of vascularized constructs with spatially patterned growth factors, which recapitulate the native capillary architecture [70]. In addition, injectable hydrogels have attracted much interest due to their capacity to fill irregular defects and undergo in situ gelation; this enables the minimally invasive delivery of cells and angiogenic factors directly to the injury site [67,80].

### 6.2. Fibrous Scaffolds (Nanofibers, Microfibers)

Fibrous scaffolds replicate the fibrous structure of the ECM, which presents both topographical and mechanical information which directs cellular alignment, migration, and differentiation. They are mainly produced by electrospinning, which makes nanofibers (50–1000 nm) or microfibers (>1 µm) of a polymeric solution under the influence of high-voltage electric fields [43].

The most common method of fabricating these scaffolds is electrospinning. It forms non-woven mats of nanofibers, which have a high surface area–volume ratio and the maximum number of surfaces to be occupied by proteins and cells, and facilitates easy diffusion of gasses and nutrients, cell adhesion, and angiogenic reactions [45]. It has been found that the topography of these nanofibers is a powerful physical stimulus; aligned fibers can cause endothelial cell elongation and the development of capillary-like tubes by contact guidance pathways [43,44]. Such an example is the use of electrospun PCL–collagen nanofibers that have been shown to improve the migration of ECs and the formation of capillary-like structures, which facilitate neovascularization in cardiac and bone tissue models [81].

Recent developments are geared towards the incorporation of microfiber in order to enhance the pore size since dense nanofiber mats may occasionally prevent extensive cellular infiltration. Biomimetic scaffolds can be developed using hybrid techniques, including gelatin in combination with electrospun PCL (polycaprolactone) fibers to form scaffolds that are mechanically stable and permit cellular invasion and formation of vessels [45,82]. Fiber diameter and orientation have a very strong effect on cell behavior—directional growth occurs on cell aligned fibers (e.g., muscle or nerve tissue)—whereas random fibers facilitate the isotropic cell dispersal, ideal in wound or skin regeneration. Moreover, core–shell electrospinning provides an opportunity to entrap growth factors or cells into the core of the fibers and release the angiogenic molecules (VEGF or bFGF) controlled over a longer time [83]. Also, by functionalizing these fibers with photothermal species, it is possible to generate multifunctional scaffolds which will not only facilitate angiogenesis but also impart therapeutic heat stimulation to promote bone regeneration [82].

### 6.3. Multi-Layered and Composite Scaffolds

Biological tissues are not homogeneous: most biological tissues are anisotropic structures with different layers (like the epidermis/dermis of the skin or the tunica layers of the blood vessel). Multi-layered scaffolds are bioinspired scaffolds designed to signify this zonal structure, offering particular guidance of various cell lines within a single structure. The layers can be customized and adapted to fulfill particular functions such as mechanical support, bioactivity and vascular integration that provide spatial control of tissue regeneration [53,58].

As an example, a bi-layered design in the context of vascular graft engineering could be to use a dense and smooth inner lining to promote an endothelial monolayer (to prevent thrombosis) and a porous outer lining to promote fibroblast infiltration and integration of the graft into the surrounding tissue [22]. Likewise, stratified scaffolds have the capacity to support chondrogenesis at the top and vascularized osteogenesis at the bottom in osteochondral tissue engineering [84]. The method has specifically excelled in skin, osteochondral, and vascular graft engineering wherein the upper layer facilitates the growth of epithelial or endothelial proliferation and the lower layer offers mechanical support [85].

Composite scaffolds can take the best properties of the classes of materials. One of the strategies is to load a soft, bioactive hydrogel (resembling the cell niche) with a stiff 3D-printed polymer frame (mechanical load-bearing capacity) [53,79]. Such hybrid architecture ensures that the scaffold will be able to resist physiological forces and yet allow a permissive microenvironment in which delicate capillary formation can take place [53]. Composite scaffolds can also be used to deliver multiple cues to the body on the same platform with cells, drugs, and growth factors in separate compartments. As an example, a 3D-printed PCL scaffold with VEGF-impregnated alginate microspheres is able to provide sustained angiogenic cues with intact mechanical integrity. Additionally, hybrid scaffolds composed of nanofibers and 3D-printed grids are more accurate in the precision of the macrostructure but, more importantly, biofunctionality at the nanoscale, spanning the difference between bulk mechanics and cellular microenvironment [86].

### 6.4. Smart Scaffolds with Responsive Properties

The second wave of bio-mimicked materials is not just about the passive assistance, but an active involvement in the healing process. The design of smart scaffolds addresses environmental responses; in response to a given environmental stimulus (pH, temperature, or enzyme concentration), the smart scaffold can change its physical characteristics or release therapeutic agents on demand. Such scaffolds have the capability to alter their properties on demand, thus increasing cellular signaling, angiogenic processes and tissue remodeling [68]. This active and adaptive behavior more closely mimics the dynamic nature of native tissue healing and regeneration.

The best example of these would be thermo-responsive hydrogels; these materials are in the form of liquid at room temperature (enabling injection), and once subjected to the normal body temperature, they are converted to a solid gel, and the defect site is filled perfectly [67]. The thermo-responsible polymer, e.g., poly (N-isopropylacrylamide) (PNIPAAm) has sol–gel transitions at the body temperature, allowing it to be injected through the smallest pore and gelled in situ to fill defects. This minimally invasive delivery approach is particularly advantageous for treating complex or deep tissue defects. Other designs take advantage of the microenvironment pathology of traumatized tissue. An example is pH-responsive scaffolds (made of chitosan or polyaniline) which swell/degrade under the conditions of acidic environment of an ischemic wound, releasing encapsulated angiogenic factors precisely when needed [68]. Such microenvironment-triggered release strategies improve therapeutic efficiency while minimizing off-target effects. Moreover, the immediate hypoxia is an essential issue to which oxygen-releasing biomaterials are addressed. Such scaffolds include oxygen-containing materials (such as calcium peroxide) that emit oxygen gradually on hydrolysis. This constant supply helps cells to survive in the deep center of the scaffold until the host blood vessels have penetrated the construct [87,88]. Further, scaffolds made of electrically conductive materials, e.g., graphene oxide, polypyrrole, or gold nanoparticles, improve cell communication and vascular morphogenesis by relaying bioelectric signals. These electroactive systems have demonstrated cardiac and neural tissue engineering applications, where the coordinated electrical activity is essential to operation [89]. Lastly, emerging “4D scaffolds” integrate smart responsiveness with time-dependent structural evolution, allowing dynamic remodeling in response to tissue growth or mechanical loading. Compared to static scaffolds, these systems can gradually adjust properties such as stiffness and degradation rate to match different stages of tissue regeneration, thereby maintaining early mechanical support while facilitating cell infiltration and vascular maturation over time. These adaptive systems represent the next generation of regenerative biomaterials, offering the potential to develop personalized, self-regulating, and vascularized tissue constructs for clinical translation [90].

## 7. Assessment of Angiogenic Performance

Assessing the angiogenic capabilities of bioinspired polymeric scaffolds is vital in determining their effectiveness to induce vascularization in vitro and in vivo. The evaluation comprises cell-based assays, animal models, and advanced imaging techniques to measure endothelial behavior, vessel formation, and functional blood perfusion in engineered tissues. A strict evaluation pipeline enables scientists to link scaffold design features such as surface chemistry, porosity, and growth factor release with physiological results, thus enhancing scaffold efficacy for clinical application. The major experimental techniques employed at each stage of this evaluation pipeline are summarized in Table 3.

### 7.1. In Vitro Evaluation Methods (e.g., Tube Formation, Cell Migration Assays)

Bioinspired scaffolds should be thoroughly in vitro tested for their cytocompatibility and angiogenic potential before going in vivo. These tests mainly utilize Human Umbilical Vein Endothelial Cells (HUVECs), which are the best source to provide a controlled and reproducible environment for studying how scaffolds influence EC proliferation, migration, and network formation [7].

One of the most common techniques is the tube formation assay, a fast-screening method recognized as the “gold standard”, where ECs are cultured on a bioinspired polymeric matrix or Matrigel to assess their network formation capability. Upon seeding on this basement membrane matrix, viable ECs perform self-assembly resulting in capillary-like networks within a few hours [91]. Scaffolds that facilitates cell adhesion and growth factor signaling normally lead to higher tube length, more branching, and network complexity [92]. To illustrate, VEGF-functionalized chitosan–PEGDA scaffolds have been demonstrated to strongly induce endothelial tubulogenesis as compared to non-functionalized samples [69]. At the same time, cell migration assays, such as the scratch wound healing assay or transwell migration assay, are used to determine the chemotactic ability of the scaffold to recruit endothelial cells, especially when the release of growth factors like VEGF is being tested [93]. Improved migration rates are usually a result of better scaffold surface topography or the installation of angiogenic peptides [62,94]. Additionally, cell viability and proliferation assays (e.g., MTT, Alamar Blue) are used for evaluating the cytocompatibility of scaffold materials, while gene and protein expression analyses (e.g., qRT-PCR, ELISA, or Western blotting) monitor the angiogenic markers’ upregulation like VEGF, FGF-2, and CD31 [81]. For scaffolds intended for vascularized or blood-contacting applications, hemocompatibility testing including hemolysis, platelet adhesion, and coagulation assays is also essential to ensure that the material does not trigger adverse thrombotic or inflammatory responses following implantation.

Traditional 2D models are limited in their capacity to reproduce the intricate 3D microenvironment of real tissue. As a result, the field is moving towards microfluidic models and “organ-on-a-chip” platforms [14]. These sophisticated setups permit the culture of ECs under normal fluid flow and shear stress and thus provide conditions to study the angiogenic sprouting as the cells respond to accurate biochemical gradients, which is closest to the real interstitial conditions in vivo [34,52]. Such 3D models give a much better forecast of how the vascular networks will develop and be maintained in the porous structure of a polymeric scaffold [14]. More complex co-culture systems with ECs and the support cells, such as fibroblasts or mesenchymal stem cells (MSCs), help in unveiling paracrine signaling and vessel stabilization mechanisms. When these are grown in polymeric hydrogels or nanofiber matrices, they reproduce the in vivo microenvironment more closely and thus reveal the interaction effects of scaffold chemistry and biomechanics on angiogenesis [95]. Although these in vitro assays are indispensable for early-stage screening, no single in vitro model can fully replicate the spatial complexity, mechanical forces, immune interactions, and perfusion dynamics of angiogenesis in vivo. Therefore, the stepwise evaluation strategy that incorporates the sequence of in vitro screening, in vivo validation, and quantitative imaging is required for the reliable prediction of scaffold performance and translation to the clinic (Table 3).

**Table 3:** Summary and comparative analysis of commonly employed in vitro, in vivo, and imaging-based techniques for evaluating cytocompatibility, angiogenic potential, and functional performance of tissue engineering scaffolds.

Category	Test Method	Technique/Principle	Key Properties Assessed	Advantages	Disadvantages	Applications	Limitations	References
In vitro	MTT/XTT/WST-1 assay	Mitochondrial metabolic activity converts tetrazolium salts into colored formazan	Cell viability, cytotoxicity	Simple, rapid, quantitative, high-throughput	Indirect measure of viability; scaffold interference possible	Initial cytocompatibility screening	Cannot distinguish proliferation vs survival, preliminary safety screening. Cannot simulate blood flow.	[81,95]
	Live/Dead staining	Fluorescent dyes label viable and non-viable cells	Cell viability, distribution	Visual confirmation, spatial information	Qualitative/semi-quantitative	Cell–scaffold interaction studies	Limited depth penetration.	[95]
	Cell adhesion assay	Quantification of attached cells after seeding	Adhesion efficiency	Simple, relevant to scaffold bioactivity	Time-dependent variability	Surface modification assessment	Does not assess long-term behavior.	[81]
	Cell proliferation assay (DNA quantification, BrdU, EdU)	Measurement of DNA synthesis or total DNA	Proliferation rate	Sensitive, quantitative	Requires cell lysis or labeling	Scaffold optimization	No functional insight.	[81]
	Tube formation assay	Endothelial self-assembly on Matrigel	Early angiogenesis	Rapid, widely accepted, direct evidence of vessel-forming ability (branching, tube length)	Lacks vessel maturation	Screening angiogenic potential	Not predictive of in vivo vasculature.	[69,92]
	Endothelial migration assay (scratch, transwell)	Measurement of directed cell migration	Migration, chemotaxis	Easy to perform	Oversimplified environment	Angiogenic signaling studies	No ECM remodeling.	[92]
	Spheroid sprouting assay	ECs spheroids embedded in ECM matrices	3D angiogenic sprouting	Mimics in vivo gradients	Lower throughput	Advanced angiogenesis studies	Complex analysis.	[95]
	Hemocompatibility tests	RBC lysis, platelet adhesion, coagulation time	Blood compatibility	Essential for vascular implants	Requires fresh blood	Vascular graft evaluation	In vitro only.	[92]
In vivo	Subcutaneous implantation (rodent)	Scaffold implanted under skin	Biocompatibility, vascular ingrowth	Simple, reproducible	Not tissue-specific	Preliminary in vivo screening	Limited physiological relevance.	[38]
	Matrigel plug assay	Angiogenic matrix implanted in vivo	Vessel infiltration	Quantifiable	Growth factor bias	Pro-angiogenic evaluation	Artificial environment.	[40,96]
	Ischemic hindlimb model	Induced ischemia in rodents	Functional angiogenesis	Clinically relevant	Technically demanding	Therapeutic angiogenesis	Ethical and cost constraints.	[2]
Imaging	Histology (H&E, Masson’s trichrome)	Tissue staining	Cell infiltration, ECM formation	Widely available	End-point analysis	Tissue integration assessment	Destructive.	[40]
	Immunohistochemistry (CD31, VEGF, α-SMA)	Antibody-based detection	Endothelial and mural cells	High specificity	Semi-quantitative	Vessel maturity analysis	Antibody dependency.	[97,98]
	Micro-CT (with contrast agents)	3D X-ray imaging	Vascular network architecture	High-resolution 3D data	Expensive, contrast needed	Quantitative angiogenesis	Radiation exposure.	[99,100]
	Fluorescence/Confocal microscopy	Optical sectioning of labeled cells	Cell distribution, tube structures	High spatial resolution	Limited penetration	In vitro and ex vivo analysis	Not whole-body.	[81]
	MRI	Magnetic resonance contrast	Perfusion, tissue integration	Non-invasive	High cost	Longitudinal studies	Low cellular resolution.	[92]
	Ultrasound Doppler	Blood flow detection	Functional perfusion	Real-time	Operator dependent	Vascular functionality	Limited microvessel detection.	[101]

### 7.2. In Vivo Animal Models for Angiogenesis Assessment

Although in vitro models can provide preliminary data, they are not capable of replicating the systemic interactions like immune response, hemodynamic pressure, and the recruitment of circulating progenitor cells that happen in a living organism. Thus, in vivo models are vital for confirming the performance of the scaffold [7]. Animal models reveal how vascular invasion, perfusion, and remodeling take place under physiological conditions.

The Chick Chorioallantoic Membrane (CAM) assay is an ideal intermediate model between in vitro studies and mammalian testing. The highly vascularized membrane of the developing chicken embryo is the site for the implantation of scaffolds to evaluate vessel convergence and infiltration. It is a low-cost method, provides easy visual monitoring, and does not require ethical approval for early-stage embryos in most areas; thus, it is a widely used method for screening bioinspired materials. CAM models are extremely effective in studying the release kinetics of angiogenic factors and inflammatory responses without the need for mammalian models [95].

For mammalian research, subcutaneous implantation in rodents (mice or rats) is the most typical model for evaluating host integration. The scaffolds are implanted dorsally, and the extent of vascular ingrowth from the host tissue is determined after certain time points (usually 1–4 weeks) [62]. Histological staining (H&E, CD31, or von Willebrand factor) is used to unveil the capillary density and vessel morphology within the scaffold matrix. Functional vascularization in specific tissue contexts can be assessed through defect models. For instance, calvarial defect models are employed as a norm in bone tissue engineering, the main purpose being to check if the vascularized scaffold is capable of supporting osteogenesis in a critical-size defect [40,96]. Similarly, myocardial infarction models are being used to figure out if cardiac patches can be of help in restoring perfusion to the ischemic heart muscle [2].

### 7.3. Imaging and Quantification Techniques

Precisely visualizing and quantifying angiogenesis are the main tasks in correlating scaffold performance with vascular outcomes. Typically, a combination of the histological, optical and molecular imaging techniques is used to evaluate vessel formation, density, and perfusion. Histological staining, for example, hematoxylin and eosin (H&E) or Masson’s trichrome, gives very detailed information about cellular infiltration, vessel morphology, and tissue integration. Immunohistochemical (IHC) staining for specific endothelial markers, such as CD31 (PECAM-1) and von Willebrand factor (vWF), enables the exact quantification of vessel density and the depiction of vessel lumen [40]. In order to measure vessel maturation, the researchers often perform double-staining to detect the pericytes (using a-SMA) that are closely associated with the endothelial channels, thus demonstrating mature and stable vasculature [56].

For volumetric visualization, the main technique is micro-computed tomography (Micro-CT), which is especially useful for bone scaffolds. By applying radiopaque contrast agents (such as Microfil), the scientists are able to map out the whole vascular tree within the scaffold and thus obtain the data on the vessel volume fraction, thickness, and connectivity [97,98]. Sophisticated optical imaging techniques, including confocal and multiphoton microscopy can be used to visualize microvascular networks inside scaffolds in three dimensions. They would be able to image the spatial arrangement of endothelial sprouts and their relationships with the microarchitecture of the scaffold [81]. Moreover, micro-computed tomography (micro-CT) with contrast agents gives a quantitative and high-resolution visualization of vascular networking’s in vivo, which can be volumetrically examined as regards vessel density, branching and perfusion.

Non-invasive imaging is used to track angiogenesis in the animal over time without sacrificing the animal. The real-time mapping of blood flow and perfusion rates with Laser Doppler Perfusion Imaging (LDPI) and ultrasound is regularly applied to give functional information about the development of the vascular network within weeks or months [99,100]. There is also the possibility of tracking labeled ECs in transparent hydrogels with recent advances in fluorescence imaging that provide information about the migration and network assembly dynamics [100].

The blood flow and oxygenation are also monitored in real time with the help of functional imaging techniques which include Doppler ultrasound, near-infrared fluorescence imaging and magnetic resonance angiography (MRA). These non-invasive devices are particularly useful in longitudinal research of the vascular remodeling process throughout the time [92]. Angiogenesis is often quantified by using image analysis programs (e.g., ImageJ, AngioTool) to quantify such parameters as total vessel length, branch points, lumen area, and perfusion index. These data combined with molecular measurements of angiogenic gene and protein expression offer a global insight into vascularization induced by the use of scaffolds [102]. A comparative summary of commonly employed in vitro, in vivo, and imaging-based techniques used to evaluate cytocompatibility, angiogenic potential, and functional vascularization of tissue engineering scaffolds is provided in Table 3.

## 8. Applications in Tissue Engineering

There is enormous potential in the application of bioinspired polymeric scaffolds in many tissue engineering processes because they can promote angiogenesis, replicate the ECM, and integrate with the host tissue, both structurally and biochemically. These biomaterials have been widely studied in bone, cardiac, neural, and skin regeneration, and in each case, sufficient vascularization is one of the prerequisites of successful repair. However, the regulatory challenges limit their clinical application despite great preclinical results. In the fabrication of scaffolds, the required material properties, the angiogenic obstacles, and the success criteria are vastly different for each tissue type, so a comparative perspective is needed.

### 8.1. Cases: Bone, Cardiac, Neural, and Skin Tissue Engineering

The vascularization requirements of engineered tissues vary significantly based on the metabolic, structural, and biomechanical requirements of tissue; thus, the design of tissue-specific scaffolds cannot be universal. Rather, the value of bioinspired polymeric scaffolds must be assessed in a tissue-specific context where angiogenic requirements are weighed against biomechanical and biological considerations. Bones require strong vascular invasion to facilitate mineralization and bone remodeling in a stiff mechanical environment, in which compressive strength and large interconnected pores are critical [69]. By contrast, heart tissue needs rapid blood flow in a compliant and electrically active environment, where scaffold compliance and electrical conductivity are as critical as angiogenic factors [29,96]. Nerve tissue is a more complex challenge where high stiffness can limit axonal regeneration, and scaffold success requires high compliance, guidance, and moderate, but stable, vascular invasion [29,83]. Finally, skin regeneration relies on accelerated angiogenesis and re-epithelialization to drive wound healing, which requires highly compliant, porous, and bioactive scaffolds that can rapidly support vascular ingrowth and wound closure [22,67].

The information provided above implies that scaffold designs should be evaluated differently in relation to their ability to develop blood vessels and the specific type of tissue being repaired, as opposed to just how well they can develop blood vessels as a general rule for all tissues combined. For example, one scaffold design may perform well when repairing bone but be too rigid when creating neural tissues; conversely, an elastic scaffold may work well when creating skin but not as well when creating bone. Therefore, when performing comparative analysis of scaffold designs, consideration must be given to both the ability to develop blood vessels and different mechanical, metabolic, and structural properties that go into determining whether a particular type of scaffold will be effective at repairing any given tissue.

Bioinspired polymeric scaffolds can be designed to meet the needs of certain anatomies due to their versatility. These applications can be represented in four major spheres:

*Bone tissue engineering*: The vascularized graft is the Holy Grail of bone regeneration because the big bone defects (critical-sized) cannot regenerate in the absence of a specific blood supply. An efficient microcirculation is needed in bone tissue to support osteogenesis, remodeling, and mineral deposition. PCL/b-TCP mixed scaffolds, collagen–hydroxyapatite hybrids, and chitosan-based matrices are examples of bioinspired scaffolds that have shown a high osteogenic and angiogenic potential because of their ability to stimulate migration and growth factor retention of ECs [70]. VEGF or BMP-2 incorporated in polymer matrices has also been demonstrated to increase microvessel infiltration and bone maturation, especially in critical-sized defects [92]. 3D-printed gradient scaffolds made of synthetic polymers and calcium phosphate also promote osteochondral regeneration due to the mechanical and biological gradient of native bone [70]. In recent strategies, 3D-printed hydroxyapatite/polymer composites have been used successfully that imitate the Haversian system of natural bone [103,104]. Indicatively, scaffolds with oxygen-releasing microparticles have been observed to preserve osteoblast viability as well as support vascular regeneration in full calvarial defects in rats [87,96]. In addition to this, sequential delivery systems containing VEGF and BMP-2 (Bone Morphogenetic Protein-2) have shown the capability of pairing angiogenesis with osteogenesis, with an enormous increase in bone volume relative to either one-factor delivery [37,66].

*Cardiac tissue engineering*: The heart contains very low regenerative activity and repairing myocardial infarction (MI) tissues is a significant issue. Bioinspired cardiac patches are aimed at giving mechanical support to the ventricular wall and promoting revascularization. Polymers that have the capacity to conduct (such as polypyrrole or aniline pentamer) have been used in both PCL and PGS scaffolds with great success in controlling the contraction speed of cardiomyocytes, whilst promoting endothelial cell growth [2,41]. According to recent research, the use of vascularized cardiac patches in rat models of MI leads to significant enhancement of left ventricular ejection fraction and scar tissue formation [2]. Formed bioinspired elastomeric scaffolds built on either PCL–gelatin or PEGDA-based hydrogels have demonstrated some potential in delivering angiogenic and cardiogenic factors directly to infarcted areas [89]. These scaffolds are capable of releasing VEGF, FGF-2, or stromal-derived factor-1 (SDF-1) in a controlled fashion to promote endothelial sprouting and neovascularization of infarcted myocardium. Electrical stimulation. Preclinical studies with electroconductive polymer composites (e.g., polypyrrole or graphene-incorporated scaffolds) have shown synchronized electrical stimulation and enhanced contractile recovery in rodent infarction models [105].

*Neural tissue engineering*: Guidance conduits are needed to regenerate axons after cutting them and to provide the metabolic requirements of the axons. Here, conductive bioinspired scaffolds play a key role, where electrical stimulation fosters neurite growth, as well as angiogenesis [106,107]. It has been established that the 3D-bioprinted nerve gusts with vascular channels undergo rapid vascularization to prevent the central necrosis of the nerve cable, resulting in a better functional recovery and myelination of the injured sciatic nerve [41,50]. Tissue regeneration in the context of the central nervous system (CNS) presents special considerations following the fact that this organ has scarce endogenous angiogenic potential. The bioinspired scaffold, specifically aligned electrospun nanofibers composed of PCL, poly-lactic acid, and fibroin silk proteins, is developed to induce axonal regeneration and, at the same time, stimulate the neovascularization of injured neural tissue [108]. The inclusion of neurotrophic and angiogenic factors, e.g., nerve growth factor (NGF) and VEGF into these scaffolds, promotes neurite outgrowth and vascular incorporation that results in better functional recovery in models of spinal cord and peripheral nerve repair [85].

*Skin tissue engineering:* Rapid neovascularization and re-epithelialization of the skin is needed to heal the barrier functions. Angiogenesis is usually disrupted in chronic wounds or diabetic ulcers. The nanofibrous dressing made by electrospinning collagen or silk fibroin resembles the dermal ECM, enhancing hemostasis and migration of cells [26]. Recently, a bioinspired silk/amniotic membrane scaffold which was also functionalized with mesenchymal stem cell-derived exosomes was created; this dressing was found to hasten the wound healing and neovascularization of diabetic mice by regulating the inflammatory response [109,110]. Collagen–chitosan-based hydrogels and electrospun PCL–gelatin nanofibers have been very effective in speeding up angiogenic and fibroblast responses during wound healing [69]. The addition of bioactive agents like honey, curcumin, and silver nanoparticles improves the antimicrobial activity as well as the vascular regeneration. Latest developments also include intelligent wound dressings that can monitor the situation in real time and release growth factors or anti-inflammatory molecules as a reaction to the surrounding conditions [111].

**Figure 2:**
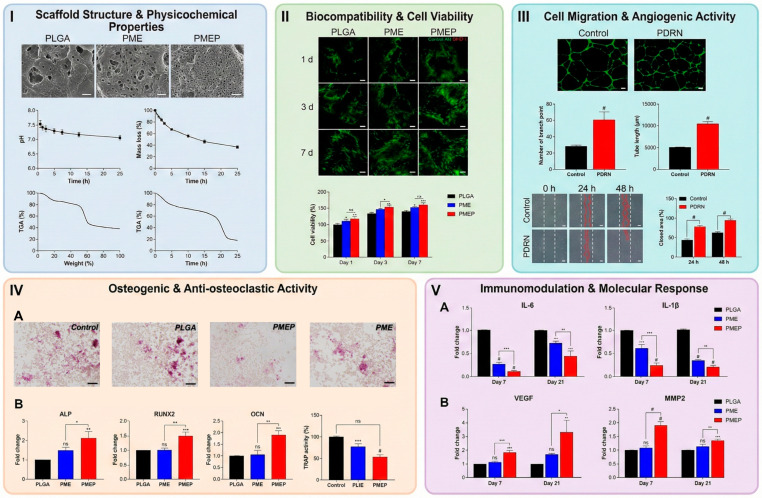
Multiscale assessment of scaffold physicochemical properties, biocompatibility, angiogenic performance, osteogenic potential, and immunomodulatory response. (**I**) Structural morphology and physicochemical characterization of the PLGA, PME, and PMEP scaffolds, including surface microarchitecture, pH dynamics, mass loss, and thermal stability; and (**II**) analysis of biocompatibility and viability of cells showing improved cellular attachment and proliferation on modified scaffolds at 1, 3 and 7 days. (**III**) Study of the migration and angiogenic activity of endothelial cells through the scratch wound healing and tube formation assay, showing the increase in branching points and tube length in PMEP-treated groups. (**IV**) Evaluation of osteogenic and anti-osteoclastic response by alkaline phosphatase (ALP) staining, osteogenic gene (ALP, RUNX2, OCN) expression, and tartrate-resistant acid phosphatase (TRAP) response, indicating enhanced osteogenesis and reduced osteoclast differentiation. (**IV**-**A**): optical images of TRAP+ cells (scale bar = 100 μm). (**IV**-**B**): gene expressions of hBMSCs related to osteogenesis onto the scaffolds; ALP, RUNX2, and OCN at 7 and 21 days of osteogenic differentiation. (**V**) Analysis of immunomodulatory and molecular responses, demonstrating the downregulation of pro-inflammatory cytokines (IL-6, IL-1v) and the upregulation of angiogenic markers (VEGF, MMP-2), which confirm the multifunctional regenerative ability of PMEP functionalized scaffolds. (**V**-**A**,**B**): Gene expressions of hBMSCs onto the scaffolds related to (**A**) anti-inflammation: IL-6 and IL-1β, and (**B**) angiogenesis: VEGF and MMP2 at 7 and 21 days. Reprinted with permission from [65] under CCBY 4.0. The differences were considered significant when ns = not significant (*p* ≥ 0.05), * *p* < 0.05, ** *p* < 0.01, *** *p* < 0.001, and # *p* < 0.0001 (n ≥ 3).

The study conducted by Kim et al. shows that poly(methoxyethyl)phosphoryl (PMEP)-modified poly(lactic-co-glycolic acid) (PLGA) scaffolds are more physicochemically stable, biocompatible, and angiogenic compared with pristine PLGA scaffolds. The scaffold structure allows for maintaining cellular viability and angiogenesis, and significantly increases capillary-like tube formation. Furthermore, PMEP-modified scaffolds promote osteogenic differentiation while simultaneously suppressing osteoclastic activity to form a favorable bone regenerative environment. Immunomodulatory profiling is a decrease in pro-inflammatory (IL-6, IL-1b) and an increase in angiogenic markers (VEGF, MMP-2). Collectively, these results highlight the potential of PMEP-functionalized polymeric scaffolds as multifunctional biomaterials that can synergistically modulate the processes of angiogenesis, osteogenesis, and immune response for the tissue regeneration application, as shown in Figure 2 [65].

**Figure 3:**
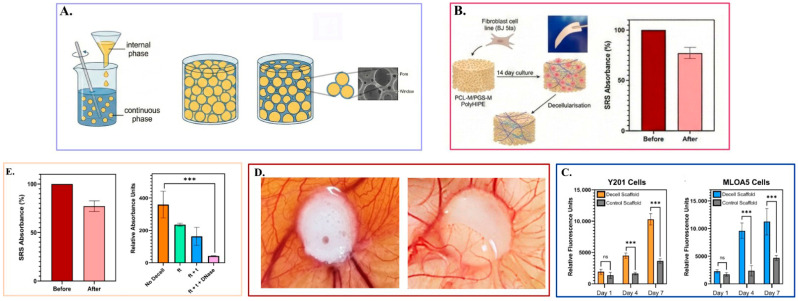
Depicts the creation and removal of cells, and testing of bioinspired polymer scaffolds for their ability to develop new blood vessels (angiogenesis). (**A**) A schematic presents how the scaffold was fabricated via emulsion templating, allowing for a well-connected network of pores in the scaffold. (**B**) The scaffold was made with fibroblast cells, which were subsequently removed through decellularization to develop scaffolds that are enriched in extracellular matrix (ECM), with quantitative measurement of the residual cellular material remaining after decellularization. (**C**) The growth of endothelial cells on the decellularized scaffold in vitro was tested against a control scaffold and shown to elicit a greater response from the endothelial cells. (**D**) The chick chorioallantois membrane (CAM) model was used to assess angiogenesis in vivo, showing substantial formation of blood vessels around the scaffold. (**E**) Quantitative analysis confirmed that the decellularization process was successful and the scaffold retained its bioactivity. Together, the results suggest that the bioinspired polymer scaffold functionalized with ECM possesses angiogenic and regenerative capacity. Reprinted with permission from [112] under CCBY 4.0. The differences were considered significant when *** *p* < 0.001.

Through a process known as emulsion templating, scientists have developed a highly porous polymeric scaffold material for biological applications that is based on a phase separation mechanism. After culturing fibroblast cells to form a three-dimensional structure and subsequently removing the cells (decellularization), there is significant evidence of cell infiltration into the scaffold and production of extracellular matrix (ECM) proteins. The resulting acellular scaffold contained high levels of ECM proteins. It has been demonstrated in vitro that the decellularized scaffold provides better potential for cellular attachment/metabolism compared to non-decellularized scaffolds. The use of the chick chorioallantois membrane (CAM) model following implantation of decellularized scaffolds indicates that there is significant vascular infiltration and angiogenesis occurring within the scaffold. In vitro studies of endothelial cells show that decellularized scaffolds have statistically significantly increased rates of proliferation versus controls. The results of these studies demonstrate that decellularized polymeric scaffolds with functionalized ECMs can support angiogenesis and regenerative healing of tissues via the vascular network, as shown in Figure 3 [112].

### 8.2. Successes and Limitations in Clinical/Preclinical Applications

Preclinical data are very consistent in showing that bioinspired polymeric scaffolds have the potential to bring about major changes in vascular infiltration, tissue integration, and functional recovery in various tissue types. For example, in animal models, hybrid scaffolds with EPCs and VEGF microspheres resulted in vascularization that was up to 60% faster, and implant survival was better by 2–3 times as compared to the use of acellular constructs [95]. Clinical-grade scaffolds such as the Integra(r) Dermal Regeneration Template (collagen-glycosaminoglycan) and Matriderm(r) have been effective in regenerating full-thickness skin with very low immunogenicity. The field has experienced a great number of preclinical successes, especially in rodent models, where bioinspired scaffolds have been shown repeatedly to have the ability to cause vascularization and regain tissue function [62,97]. The use of advanced technologies like 3D bioprinting has made it possible to produce clinically relevant construct sizes that were previously unsustainable [49,75]. Importantly, 3D bioprinting additionally allows precise spatial patterning of multiple cell types, growth factors, and vascular architectures within a single construct, a level of organizational control that is challenging to attain using conventional scaffold fabrication methods [113].

Several factors are obstructing the transfer of these achievements to human applications. A major limitation is the scalability of these complicated manufacturing processes; producing a custom, drug-eluting, nanofibrous scaffold under GMP (Good Manufacturing Practice) conditions is expensive and requires a high level of technical skill [14,36]. Synthetic scaffolds usually need additional surface biofunctionalization to provide enough cell adhesion and vascularization. Besides that, the immune response to polymer degradation byproducts and the possibility of infection or fibrosis may lead to the problem of graft integration [114]. Regulatory challenges, such as the requirement for long-term safety confirmation and standardized manufacturing protocols, are among the reasons why clinical translation is still at a standstill. In addition, although small animal models are promising, they often cannot predict the immune response in humans. The “foreign body response” to synthetic polymers may result in fibrous encapsulation, which physically hinders the penetration of new blood vessels, thereby causing graft failure [60]. Moreover, the issue of long-term safety of degradation byproducts, especially from novel smart polymers, is still a regulatory obstacle that necessitates extensive longitudinal studies [14,115]. In spite of these drawbacks, the ongoing work that combines 3D bioprinting, stem cell technologies, and smart biomaterials is gradually bridging the gap between preclinical success and clinical applicability. In particular, hybrid bioprinted constructs incorporating predefined vascular channels and controlled drug-release functionalities are increasingly viewed as promising approaches for clinical translation, and several such systems are currently undergoing the feasibility evaluation phase in early-stage clinical trials for bone and soft tissue reconstruction [70].

### 8.3. Patent Updates

Recent patent activities (2020–2025) indicate a significant movement toward hybrid biomaterials (Table 4). Most of the inventions are about composite scaffolds that merge natural and synthetic polymers like PCL–collagen or PEG–alginate to maintain biocompatibility while also providing mechanical strength. The designs reveal a substantial focus on vascularization since they incorporate VEGF delivery systems, microchannels, or peptide functionalization to facilitate angiogenic sprouting and endothelial cell migration. At the same time, integration of smart technologies is getting more and more attention as quite a few patents deal with the scaffolds that can respond to pH changes, mechanical stress, or bioelectric cues and represent a step forward in the intelligent regenerative biomaterials. Importantly, a few of the patented systems are moving towards the market, thus showing scalability and compliance with GMP standards and, therefore, revealing an increase in regulatory readiness and clinical translation potential.

As the science moves closer to becoming a feasible technology, the intellectual property (IP) environment for bioinspired scaffolds has broadened. Presently, patent activity is mainly focused on novel fabrication methods and bioink formulations. Recent publications highlight that the main area of protection for proprietary bioinks is the ones that facilitate printability and cell viability for 3D bioprinting vascular networks. One of the essential innovations for the commercialization of vascularized tissues is “sacrificial inks” that dissolve to leave behind hollow channels [49,75]. Another key focus is the functionalization of peptide sequences, as an increasing number of patents target short-chain peptides, such as the QK peptide (106), which mimic the bioactive domains of growth factors. Such synthetic substitutes are more stable and easier to patent than the natural proteins [68]. Besides that, intelligent, responsive material systems (for example, pH-triggered hydrogels) are experiencing a significant increase in IP filings as they represent a separate class of “medical devices” with superior therapeutic utility [67]. The move towards the protection of these particular material compositions and production methods indicates a change in the focus from academic discovery to commercial product development [10].

### 8.4. Regulatory Concerns and Commercially Available Scaffold Products

Scaffolds, which are normally classified as a medical device, have to go through several levels of regulation before they can be available for use in a clinical setting. These regulations help to ensure that each type of device meets all safety, efficacy, and performance requirements, and minimize risks associated with using them in humans. For example, in the US, the FDA divides devices into three Classes I (low risk); II (moderate risk, requires 510(k) clearance and substantial equivalence to other similar devices, i.e., predicate devices in the market) and III (high risk, requires Pre-Market Approval (PMA) based on evidence from clinical trials to establish safety and effectiveness.) A de novo classification route exists, allowing for novel scaffolds to be classified into lower risk classes without a predicate device if appropriate controls can be established. However, some biologically based scaffolds may also be considered human cells, tissues, and cellular/tissue-based products (HCT/Ps). In addition, in the European Union (EU), all scaffolds fall under the Medical Device Regulation (EU MDR 2017/745), which requires that the device be placed into the EU CE marked system before marketing in any country within the EU. Therefore, relatively straightforward scaffolds made from familiar synthetic or naturally occurring polymers tend to achieve faster time-to-market compared to more complex bioinspired designs such as hybrid designs or growth factor-loaded scaffolds, which tend to be Class III or combination products that will require substantial clinical testing before being marketed and, therefore, will have longer development cycles.

Few scaffold technologies for both regenerative medicine and wound healing have made it to routine clinical use. Scaffolds primarily used in skin and soft tissue regeneration have fewer regulatory barriers, and their clinical endpoints are generally clearer than for more complex engineering applications. Some examples of scaffolds that have been used for these purposes include Integra Dermal Regeneration Template (a bilayer collagen and glycosaminoglycan scaffold that FDA approved through the PMA process for the reconstruction of burns and wounds), Apligraf (a cellular scaffold made from polymer material that is indicated for the treatment of chronic wounds), and Dermagraft (a polymer-based cellular scaffold that is indicated for the treatment of diabetic ulcers). These examples illustrate the regulatory and commercial success of scaffolds used in biomaterial-based repair and regeneration, but they are relatively simple designs. Commercial success in the development of these products is very dependent on the use of biocompatible and well-characterized biomaterials such as collagen and PLGA (poly-lactic-glycolic acid) to ensure that they are both safe and scalable, which, in turn, suggests that there is significant room for improved translation of more complex preclinical scaffolds into clinically relevant products, as shown in Table 5.

## 9. Challenges and Future Perspectives

Although the design of bioinspired polymeric scaffolds in angiogenesis and tissue engineering has made impressive progress, there are still a few scientific, manufacturing, and translational threats to clinical practice. These constraints and future opportunities that have been outlined can help develop these materials in the laboratory into reproducible, safe, and scalable therapeutic solutions.

### 9.1. Limitations in Current Bioinspired Scaffold Designs

Even though bioinspired scaffolds can faithfully mimic the ECM, most of the existing scaffold designs do not achieve the full complex mechanical, biochemical, and dynamic characteristics of the native tissues. A significant drawback is the fact that multiscale ECM hierarchies remain incompletely mimicked, especially in thick, vascularized tissues such as myocardium or bone, where spatial heterogeneity in stiffness, fiber orientation, and biochemical gradients are important in cellular signaling [116]. The first problem is the trade-off between mechanical strength and porosity. Highly porous scaffolds needed to allow rapid vascular infiltration are often not mechanically resistant to allow load-bearing tissues such as bone, and mechanically resistant scaffolds are often too dense to allow endothelial cell migration [10]. Moreover, the majority of existing scaffolds are inert structures, whereas the native ECM is a dynamic microenvironment, which undergoes constant remodeling based on physiological signals. Despite advances in smart materials, precise long-term spatiotemporal control of growth factor releases that mimic the complex cascade of natural wound healing remains elusive [66,106]. Another crucial problem is the immunogenicity. Synthetic polymers will result in the development of a chronic foreign body reaction, which results in fibrotic envelopment and isolates the scaffold of the host vasculature. Even natural scaffolds with the decellularization method have the risk of remaining DNA or antigens to induce immune rejection [15,56].

Some natural polymers like collagen or chitosan can also be highly biocompatible, but their batch-to-batch reproducibility and lack of control over their degradation kinetics make them difficult to reproduce and mechanically unstable. By comparison, synthetic polymers such as PCL or PLA offer structural stability, but they do not have cell-recognition motifs, and they typically need functionalizing after fabrication [85]. Moreover, it is hard to regulate the release of angiogenic cues. Many scaffolds show an initial burst release of VEGF or bFGF, resulting in insufficient spatial–temporal control of angiogenesis and untimely depletion of bioactive agents [69].

### 9.2. Scalability, Reproducibility, and Regulatory Obstacles

The barrier to the realization of prototypes in commercial products is high levels of manufacturing and regulatory hurdles. Existing processes of fabrication, like electrospinning, freeze-drying, or solvent casting, have not been easy to scale and retain microstructure precision and reproducibility. This limitation is particularly pronounced for bioinspired and vascularized scaffolds, where small deviations in architecture can significantly affect biological performance. Methods such as electrospinning and high-resolution 3D bioprinting are typically slow and cannot be used to develop scaffolds on a large scale without damaging the fragile nano-architecture or biological functionality of the scaffold [14,36].

Differences in the angiogenic response to batches of polymer molecular weight, degradation rate, and pore geometry may occur due to batch variability in polymer molecular weight, degradation rate, and pore geometry, which are non-conforming under regulatory requirements, such as the 21 CFR Part 820 of the U.S. FDA or the European Medical Device Regulation (EU MDR 2017/745). Such batch-to-batch variability poses a major challenge for standardization and quality control in clinical-grade scaffold production. In addition, storage stability and sterilization are significant issues. Numerous bioinspired polymers can be degraded or lose bioactivity by gamma radiation or ethylene oxide sterilization. As an example, PEG-based hydrogel and collagen matrices tend to have their crosslinking density or biofactor denaturation altered after sterilization.

The hybrid characteristics of bioinspired scaffolds are also a complication of regulatory approval processes because they can involve both biological molecules, living cells, or drugs, and are considered combination products. The individual components (e.g., VEGF, polymer matrix, or stem cells) will have to ensure different biocompatibility and toxicity parameters, and, hence, preclinical validation has been proven to be incredibly expensive and time-consuming. It also requires large-scale, Good Manufacturing Practice-compliant production systems, which many research institutions simply do not have [95].

### 9.3. Future Directions: Integration with Advanced Technologies

The future of vascularized tissue engineering is in the combination of material science with the best fabrication and computer technologies. The current trends of bioinspired scaffold design shift towards multifunctional, smart, and highly personalized regenerative systems through the combination of bioprinting, nanotechnology, artificial intelligence, and dynamic biomaterials.

The next stage is the 4D bioprinting that brings in the fourth dimension, which is time. The morphing scaffolds of these kinds can also dynamically change their geometry after implantation, responding to physiological cues (e.g., temperature or humidity), mechanically stimulating the tissue surrounding the constructs to induce vascular alignment [67,75]. Such time-dependent behavior offers new possibilities for guiding vascular maturation and tissue remodeling in vivo. 3D and 4D bioprinting will transform the manufacture of scaffolds by allowing the construction of patient-specific geometries featuring vascular channel networks that have been precisely positioned by constructs. This level of spatial and temporal control is difficult to achieve using conventional fabrication techniques. Micro-extrusion and stereolithography-based bioprinting can be used to deposit polymers, hydrogels, and ECs at the same time to produce pre-vascularized tissue patches, whereas 4D bioprinting goes a step further to develop shape-, stiffness-, or porosity-changing scaffolds in response to mechanical or biochemical stimuli [70]. Together, these advances are expected to play a key role in overcoming current translational barriers in vascularized tissue engineering.

Nanotechnology will be relevant in polishing delivery systems. It is probable that future scaffolds will be able to use intelligent nanomaterials with the ability to detect the immediate environment including the presence of high levels of reactive oxygen species in ischemic tissue and can then respond by releasing antioxidant or angiogenic cargo to that specific location [13,117]. Improved cell adhesion, electrical conductivity, and local angiogenic factor delivery is enabled by incorporation of nanoparticles (e.g., gold, silica, or hydroxyapatite) and nanoscale topographies. Hierarchical nanofibrous scaffolds are also more closely aligned with the ECM that guides endothelial alignment and vascular morphogenesis. Another potential frontier is the combination of AI and computational modeling in scaffold design. Predictive models have the ability to simulate the cell–material interaction, angiogenic diffusion field, and mechanical stress fields, and speed up the optimization of scaffold geometries to be optimized prior to fabrication. The AI-based design-to-fabrication systems will offer entirely personalized scaffolds that will be specific to the anatomy and pathophysiology of the patient [11,13].

## 10. Conclusions

As bioinspired polymeric scaffolds continue to develop towards creating fully vascularized and functionalized tissue constructs, replicating the structural hierarchy, biochemical signaling and dynamic responsiveness of the native ECM has opened up a wide range of opportunities for promoting angiogenesis and tissue regeneration in various organ systems. Advances in polymer chemistry and fabrication have resulted in hybrid scaffolding materials that combine the essential characteristics of mechanical strength, biocompatibility and tunable biodegradability, critical to achieving clinical success over an extended time period. The incorporation of controlled delivery systems, biomimetic surface chemistries and pre-vascularization strategies to include angiogenic cues into hybrid scaffold systems have been effective in promoting measurable improvements in vascular integration, as demonstrated through preclinical and clinical studies. The translation of these materials from laboratory to clinic remains challenging due to ineffective production processes, regulatory issues, and biocompatibility issues. Having the ability to produce hybrid scaffolds with equal architecture and a continuous bioactive agent delivery is essential for success. Another significant factor that must be addressed is the evaluation of scaffold immune compatibility in order to facilitate successful clinical applications. Addressing these problems is critical to ensure the long-term success of hybrid scaffolds in the field of healthcare. Future developments will allow for the utilization of intelligent biomaterials—hybrid scaffolds capable of sensing and responding to their local microenvironment, fabricated using 4D bioprinting technology, and optimized through artificial intelligence-driven computational modeling. There is little doubt that the integration of nanotechnology will greatly enhance the ability to control the angiogenic signaling process on the molecular level. It is expected that the combined power of these technologies will revolutionize the development paradigm.

## Figures and Tables

**Table 1:** Ranking of scaffold requirements for angiogenesis and tissue regeneration.

Tier	Requirement Category	Priority Level	Consequence of Insufficiency	Ref(s)
I	Safety and Biocompatibility	Absolute prerequisite	Acute/chronic inflammation, implant rejection, patient harm	[37,38]
II	Porosity and Interconnectivity	Critical for survival	Central necrosis, inadequate nutrient/waste exchange, cell death	[8,10,12]
III	Biodegradability and Mechanical Integrity	Essential for function	Premature collapse vs. chronic foreign body reaction, impaired regeneration	[19,30]
IV	Surface Bioactivity and Advanced Cues	Important for optimization	Slower/poorer vascularization; may still permit some healing	[39,40,41]

**Table 2:** Comparative mechanical property requirements for scaffolds targeting different tissue types.

Tissue Type	Compressive Strength (MPa)	Tensile Strength (MPa)	Elastic Modulus (MPa)	Elongation at Break (%)	Key Mechano-Transudative Effects	Ref(s)
Cortical bone	100–150 (native: 100–200)	50–100	10,000–20,000	1–3	Promotes osteogenic differentiation via YAP/TAZ signaling	[27,28]
Cancellous bone	2–12 (native: 2–12)	5–15	100–1000	5–10	Directs MSC differentiation toward osteogenic lineage	[27,28]
Cartilage	1–10	5–25	10–100	15–30	Maintains chondrocytic phenotype; resists compressive loading	[27,30]
Cardiac muscle	0.05–0.5	2–15	0.5–5 (native: 0.1–1 diastolic)	20–40	Regulates cardiomyocyte contractile function and gap junction formation	[29,40]
Skeletal muscle	0.1–1	10–40	10–50	20–40	Promotes myotube alignment and fusion	[27]
Skin (dermis)	0.1–0.5	2–30 (native: 2–20)	0.4–0.8	30–70	Supports fibroblast-to-myofibroblast differentiation	[22,27]
Vascular (artery)	0.1–0.5 (radial)	1–10 (circumferential: 5–15)	0.1–1 (native: 0.5–5)	50–150	Promotes endothelial alignment and smooth muscle cell phenotype regulation	[21]
Neural (peripheral nerve)	0.01–0.1	0.5–5	1–10	30–50	Guides neurite extension via contact guidance	[40]
Neural (brain/CNS)	0.001–0.01	0.1–0.5	0.1–1	N/A	Maintains neural stem cell phenotype; limits glial scarring	[41,42] https://doi.org/10.1016/j.biomaterials.2009.09.002

**Table 4:** Summary of recent patents related to bioinspired polymeric scaffolds for angiogenesis and tissue engineering applications.

Patent Number	Title/Year	Key Invention Focus	Relevance to Angiogenesis and Tissue Engineering
US11246961B2	Engineered scaffolds for vascularized tissue repair (2022)	Cyclic peptide-functionalized polymer scaffolds for endothelial adhesion and vascular ingrowth.	Enhances endothelial cell recruitment, capillary formation, and scaffold vascularization.
US20210322635A1	Tissue scaffold with synthetic and natural polymers (2021)	Hybrid composite scaffolds integrating biodegradable synthetic and natural polymers.	Supports angiogenesis and regeneration in soft and hard tissue constructs.
EP3651817B1	Biodegradable elastin-based tissue scaffold (2023)	Elastin-inspired polymeric scaffold with controlled degradation and porosity.	Promotes nutrient and vascular infiltration for improved tissue integration.
US7575759B2	Tissue engineering scaffolds with controlled porosity (2009)	Porogen fusion process for uniform pore size and interconnectivity.	Improves oxygen and nutrient transport, facilitating vessel ingrowth.
US8758781B2	Biological scaffolds for promoting vascularization (2014)	Collagen-based laminate scaffolds with bioactive coatings.	Designed for wound healing and enhanced angiogenic response.
US10137184B2	Scaffold with bioactive coatings for cell transplantation (2018)	Incorporates adhesion ligands and ECM peptides in polymer scaffolds.	Promotes endothelial cell migration and angiogenic factor expression.
US8197743B2	Hydrogel constructs via stereolithography (2012)	Layer-by-layer printed hydrogel scaffolds with lumen-like channels.	Enables patterned vascular channels and controlled growth factor localization.
EP3924008NWB1	Vascularizing devices and methods for implanted tissue constructs (2024)	Bioengineered scaffolds with endothelialized channels and hydrogel matrices.	Accelerates perfusion and pre-vascularization in implantable constructs.
US6596296B1	Drug-releasing biodegradable fiber implants (2003)	Biodegradable polymeric fibers for sustained release of therapeutic molecules.	Enables controlled delivery of VEGF or angiogenic drugs at tissue sites.
US7923486B	Biopolymer scaffold-sheet methods for tissue engineering (2011)	Cross-linked elastomeric scaffold sheets from biopolymers.	Provides flexible ECM-mimicking support for vascularized tissue repair.
US9707322B2	Gradient porous scaffold for tissue regeneration (2017)	Scaffold with varying pore gradients fabricated via additive manufacturing.	Supports vascular infiltration and hierarchical tissue growth.
US8642336B2	Microchannel-embedded scaffold for vascular networks (2014)	Scaffold microarchitecture mimics natural vascular geometry.	Facilitates perfusion and pre-vascularization within thick tissues.
WO2022231194A1	Vegf-loaded PCL–collagen composite scaffold for bone repair (2022)	Dual-phase PCL/collagen structure with sustained VEGF release.	Induces angiogenesis and osteogenesis synergistically in bone defects.
US20230147789A1	Electrospun scaffolds functionalized with nitric oxide donors (2023)	Electrospun nanofibers releasing nitric oxide for endothelial activation.	Promotes endothelialization and prevents thrombosis in vascular grafts.
EP4039917B1	4D-printed bioresponsive scaffolds (2024)	Shape-morphing polymer scaffolds responsive to mechanical or pH stimuli.	Supports adaptive vascular remodeling during healing.

**Table 5:** Comparative overview of commercially available and clinically approved polymeric scaffold systems for tissue engineering.

Tissue Category	Product Name	Manufacturer	Material Composition	Regulatory Status (FDA/CE-Mark)	Key Clinical Indications	Ref
Skin (dermal templates)	Integra^®^ Dermal Regeneration Template	Integra LifeSciences (NJ-USA)	Bovine tendon collagen + shark chondroitin-6-sulfate + outer silicone layer (bilaminar)	FDA PMA (1996); CE-Mark	Full-thickness burns, traumatic wounds, diabetic foot ulcers, scar revision; forms neodermis, facilitates vascular infiltration within 2–4 weeks	[11,14]
	Matriderm	MedSkin Solutions Dr. Suwelack AG (Billerbeck-Germany)	Type I, III, V collagen + elastin (single layer)	CE-Mark (2005); FDA 510(k)	Full-thickness burns, traumatic wounds; single-stage application with split-thickness skin graft (no delayed second surgery)	[14]
	PELNAC^®^ (Pelnac^®^ Bilayer Wound Matrix)	Gunze (Kyoto-Japan)	Bilayer wound matrix (collagen-based)	FDA 510(k) (2020); CE-Mark; PMDA (Japan, 1993)	Burns, traumatic skin defects, post-oncologic defects; marketed in Europe, the Middle East, South Africa, China, Korea, Brazil	[33]
	NovoSorb^®^ Biodegradable Temporizing Matrix (BTM)	PolyNovo (Victoria-Australia)	Synthetic biodegradable polyurethane (bilaminar)	FDA 510(k) (2025); CE-Mark (earlier)	Complex wounds, full-thickness burns, pressure ulcers, diabetic ulcers; synthetic, non-animal-derived, support dermal regeneration within 2–4 weeks	[14,22]
	PermeaDerm^®^	Stedical Scientific (CA-USA)	Biosynthetic wound matrix	FDA 510(k) (K153678)	Partial-thickness wounds, surgical wounds, trauma wounds, donor sites	[36]
Peripheral Nerve	Neurotube^®^	Synovis (Alabama-USA)	Polyglycolic acid (PGA) mesh tube	FDA 510(k) (K983007, 1999)	Peripheral nerve gaps ≥8 mm to ≤3 cm; first biodegradable nerve conduit cleared in the US	[95]
	Neuroflex™	Collagen Matrix, Inc. (NJ-USA)	Type I collagen (semipermeable, flexible, tubular)	FDA cleared (2001)	Hand surgery, nerve reconstruction, painful neuromas; 4–8-month degradation	[83]
	NeuraGen^®^/NeuroMatrix™	Collagen Matrix, Inc. (NJ-USA)	Type I collagen	FDA cleared; CE-Mark	Peripheral nerve repair	[85]
	Neurolac^®^	Polyganics (Groningen-Netherlands)	PLCL (poly(DL-lactide-ε-caprolactone))	CE-Mark; FDA cleared	Peripheral nerve gaps; biodegradable synthetic conduit	[85]
	Avance^®^ Nerve Graft	Axogen (Florida-USA)	Decellularized human nerve allograft (off-the-shelf)	FDA cleared	Peripheral nerve gaps up to 70 mm; processed human tissue	[90]
Bone/Orthopedic	Infuse^®^ Bone Graft	Medtronic (Minneapolis-USA)	rhBMP-2 on absorbable collagen sponge	FDA PMA (bone graft indication)	Spinal fusion, tibial fractures, osteoinductive growth factor therapy	[59]
	InductOs^®^	Pfizer (Surrey-UK)	rhBMP-2 on collagen scaffold	CE-Mark	Acute tibial fractures, spinal fusion	[59]
	Osteopore Osteoplug^®^/Osteomesh^®^	Osteopore (Singapore)	3D-printed polycaprolactone (PCL) resorbable scaffold	FDA 510(k); CE-Mark	Craniomaxillofacial defects, burr hole repair	[1]
	Cerament^®^ (BVF, G, V)	Bonesupport (Sweden)	Injectable bioceramic bone graft substitute (remodels to host bone, drug-eluting capability)	FDA cleared; CE-Mark; proposed NTAP reimbursement (2026)	Fractures, bone voids, osteomyelitis, revision arthroplasty, and infected diabetic foot	[74]
	Dimension Inx CMFlex^®^	Dimension Inx (IL-USA)	3D-extruded calcium phosphate (CaP) composite	FDA 510(k) (2023)	Bone defect filling and shaping	[1]
	3D Systems VSP^®^ PEEK/Metal CMF	3D Systems (South Carolina-USA)	PEEK and metal printing	FDA 510(k) (2024)	Custom craniomaxillofacial reconstruction	[1]
Cardiovascular	PeriBeam^®^ Pericardial Membrane	Tama Bio (Tokyo-Japan)	ePTFE (expanded polytetrafluoroethylene) with ion-beam irradiation	FDA 510(k) (K240775, Class II); PMDA (Japan, 2018, Class IV)	Pericardial patch, intracardiac defect repair; high biocompatibility, reduced adhesion, and infection risk	[65,110]

## Data Availability

No new data were created.

## Abbreviations

The following abbreviations are used in this manuscript: **α-SMA**—Alpha Smooth Muscle Actin; **ADME**—Absorption, Distribution, Metabolism and Excretion; **AI**—Artificial Intelligence; **ALP**—Alkaline Phosphatase; **bFGF**—Basic Fibroblast Growth Factor; **BMP-2**—Bone Morphogenetic Protein-2; **CAM**—Chick Chorioallantoic Membrane; **CD31**—Cluster of Differentiation 31 (PECAM-1); **CNS**—Central Nervous System; **CT**—Computed Tomography; **dECM**—Decellularized Extracellular Matrix; **EC**—Endothelial Cell; **ECM**—Extracellular Matrix; **EPCs**—Endothelial Progenitor Cells; **FDM**—Fused Deposition Modeling; **FGF-2**—Fibroblast Growth Factor-2; **GMP**—Good Manufacturing Practice; **H&E**—Hematoxylin and Eosin; **HIF-1α**—Hypoxia-Inducible Factor-1 alpha; **HUVECs**—Human Umbilical Vein Endothelial Cells; **IHC**—Immunohistochemistry; **IL-1β**—Interleukin-1 Beta; **IL-6**—Interleukin-6; **IVIVC**—In Vitro–In Vivo Correlation; **LDPI**—Laser Doppler Perfusion Imaging; **MAPK**—Mitogen-Activated Protein Kinase; **MI**—Myocardial Infarction; **MMP-2**—Matrix Metalloproteinase-2; **MRI**—Magnetic Resonance Imaging; **MSCs**—Mesenchymal Stem Cells; **NDDS**—Novel Drug Delivery Systems; **NGF**—Nerve Growth Factor; **OCN**—Osteocalcin; **PCL**—Polycaprolactone; **PDGF**—Platelet-Derived Growth Factor; **PEG**—Polyethylene Glycol; **PEGDA**—Polyethylene Glycol Diacrylate; **PECAM-1**—Platelet Endothelial Cell Adhesion Molecule-1; **PGS**—Poly(glycerol sebacate); **PLA**—Poly-lactic Acid; **PLGA**—Poly(lactic-co-glycolic acid); **PNIPAAm**—Poly(N-isopropylacrylamide); **PVA**—Polyvinyl Alcohol; **QK peptide**—VEGF-mimetic peptide sequence; **RGD**—Arginine–Glycine–Aspartic Acid; **ROS**—Reactive Oxygen Species; **RUNX2**—Runt-Related Transcription Factor-2; **SDF-1**—Stromal Cell-Derived Factor-1; **SLA**—Stereolithography; **SMCs**—Smooth Muscle Cells; **TE**—Tissue Engineering; **TIPS**—Thermally Induced Phase Separation; **TRAP**—Tartrate-Resistant Acid Phosphatase; **VEGF**—Vascular Endothelial Growth Factor; **vWF**—von Willebrand Facto.
